# Integrated halide perovskite photoelectrochemical cells with solar-driven water-splitting efficiency of 20.8%

**DOI:** 10.1038/s41467-023-39290-y

**Published:** 2023-06-26

**Authors:** Austin M. K. Fehr, Ayush Agrawal, Faiz Mandani, Christian L. Conrad, Qi Jiang, So Yeon Park, Olivia Alley, Bor Li, Siraj Sidhik, Isaac Metcalf, Christopher Botello, James L. Young, Jacky Even, Jean Christophe Blancon, Todd G. Deutsch, Kai Zhu, Steve Albrecht, Francesca M. Toma, Michael Wong, Aditya D. Mohite

**Affiliations:** 1 Department of Chemical and Biomolecular Engineering, Rice University, Houston, Texas 77005 USA; 2 Chemistry and Nanoscience Center, National Renewable Energy Laboratory, Golden, Colorado 80401 USA; 3 Chemical Sciences Division, Lawrence Berkeley National Laboratory, Berkeley, CA USA; 4 Young Investigator Group Perovskite Tandem Solar Cells, Helmholtz-Zentrum Berlin, 12489 Berlin, Germany; 5 Material Science and Nanoengineering, Rice University, Houston, Texas 77005 USA; 6 Univ Rennes, INSA Rennes, CNRS, Institut FOTON, UMR 6082, Rennes, F-35000 France

**Keywords:** Electrocatalysis, Hydrogen energy, Electrocatalysis, Photocatalysis, Photocatalysis

## Abstract

Achieving high solar-to-hydrogen (STH) efficiency concomitant with long-term durability using low-cost, scalable photo-absorbers is a long-standing challenge. Here we report the design and fabrication of a conductive adhesive-barrier (CAB) that translates >99% of photoelectric power to chemical reactions. The CAB enables halide perovskite-based photoelectrochemical cells with two different architectures that exhibit record STH efficiencies. The first, a co-planar photocathode-photoanode architecture, achieved an STH efficiency of 13.4% and 16.3 h to t_60_, solely limited by the hygroscopic hole transport layer in the n-i-p device. The second was formed using a monolithic stacked silicon-perovskite tandem, with a peak STH efficiency of 20.8% and 102 h of continuous operation before t_60_ under AM 1.5G illumination. These advances will lead to efficient, durable, and low-cost solar-driven water-splitting technology with multifunctional barriers.

## Abstract (web summary)

High-efficiency photoelectrodes, which integrate light absorption with catalysis, have been limited to costly materials. Here, the authors develop an anticorrosion barrier that enables low-cost semiconductors for integrated solar fuel devices with 20.8% solar-to-hydrogen energy conversion efficiency.

## Introduction

The manufacture of valuable products and fuels using available, low-cost feedstocks like water, carbon dioxide, and nitrogen, and solar energy is a promising way to decarbonize industry and facilitate clean energy storage. Splitting water at 25 °C requires a thermodynamic minimum of 1.23 V, but the kinetics of oxygen and hydrogen evolution reactions limit the efficiency by raising the minimum practical potential difference to 1.6 V for state-of-the-art catalysts. This large voltage requirement restricts the choice of suitable semiconductor and photovoltaic device material and design options. Several strategies for sunlight-driven, electrochemically mediated green hydrogen generation have emerged^1–6^. Comparison of these demonstrations has relied primarily on two key figures of merit: stability (lifetime at a fraction of initial efficiency) and solar-to-hydrogen (STH) efficiency, which is defined as:

1 $${{{{{\rm{STH}}}}}}=\frac{1.23{{{{{\rm{V}}}}}}\times {J}_{{op}}\times {{{{{\rm{FE}}}}}}}{{P}_{{sunlight}}}$$

where J_op_ is the operating current density, FE is the Faradaic efficiency, and P_sunlight_ is the incident power density of illumination. Integrated photoelectrochemical (PEC) devices with buried junctions, wherein the photo-absorber is protected by a barrier material modified to have a catalytically active surface, offer a high device lifetime while minimizing cost. Furthermore, coupling photo-absorbers directly to catalysts and improving solar energy utilization through thermal integration can efficiently facilitate electrochemical reactions and simultaneously cool the photovoltaics to mitigate thermal degradation and enhance their long-term durability^7^. Through judicious choice of materials and design as in this work, each component in an integrated PEC (photo-absorber, barrier, and catalysts) can even retain a substantial degree of modularity, allowing independent production and assembly at point-of-use. These advantages enable reductions in balance-of-systems costs compared with decoupled photovoltaic-electrolyzer systems. State-of-the-art PEC devices have used III-V semiconductor-based multijunction photovoltaic devices protected by thin coatings of titania and decorated with hydrogen or oxygen evolution electrocatalysts, to achieve STH efficiencies exceeding 19% with 2 h of unassisted operation for a device with up to 0.3 cm^2^ active area^8^. Greater stability has been demonstrated, up to 100 h, but at a lower STH efficiency of 10%^9,10^. Despite their promising STH efficiency, conventional III-V-based PEC platforms are potentially limited by the use of cost prohibitive semiconductors produced using molecular beam epitaxy and also due to their high propensity to undergo photo-corrosion in acidic electrolyte^11–13^. PECs based on other semiconductors such as metal oxides (e.g., BiVO_4_, hematite, and copper oxide) offer scalable and low-cost solutions but exhibit STH efficiencies well below 10% and overall low stabilities^6,14,15^.

Recently, halide perovskites (HaP) have emerged as low-cost solution-processed semiconductors, with several desirable properties such as large absorption coefficients, tunable bandgaps, long diffusion lengths, and charge-carrier lifetimes which have enabled power conversion efficiencies (PCE) exceeding 25% for single-junction perovskite solar cells (PSCs)^16,17^. The record efficiencies in single-junction HaP photovoltaic devices have been enabled by the greater than 1.0 V open-circuit voltage generated by an archetypical HaP with a bandgap of 1.5 eV. Importantly, PSCs can be combined in series with another HaP or silicon to produce an operating voltage exceeding 1.7 V, which can surmount the potential required for unassisted water splitting at high current densities^18^. However, to realize an integrated halide perovskite photoelectrochemical device (HaP-PEC), in which at least one interface is in direct contact with the electrolyte, requires preventing the spontaneous dissolution of halide perovskites in an aqueous environment due to their ionic character^19,20^. Previous attempts using HaP-PECs have pursued two main approaches. One is to tailor the surface of the HaP and the transport layers through the deposition of ultrathin surface barriers/catalysts such as Ni or titania, or by introducing hydrophobic functional groups and dopants to the transport layers^21–23^. The other common approach is to deposit or mechanically attach physical barriers (low melting point metal alloys, conducting inks, and/or mechanically attached conductive sheets such as metal foil or graphite) directly on the photovoltaic device^15,24–30^. While these strategies can in some cases preserve the short-circuit current density, all reported approaches have resulted in a loss in fill factor and the operating voltage at the maximum power point has limited their capacity for unassisted operation. As a result, the state-of-the-art STH efficiency for integrated co-planar HaP-PEC systems reported sits at about 10.6%^31^, which is only about 40% of the state of the art in perovskite solar cell PCE. A mandatory advancement for building an efficient and stable integrated HaP-PEC is a lossless anticorrosion barrier, which protects the photo-absorber and transport layers from the corrosive electrolytes without compromising the electronic transport of photogenerated electrons or holes from the HaP to the catalyst deposited on the barrier.

## Results

Here, we report the design and fabrication of an anticorrosion barrier referred to as a conductive adhesive-barrier or CAB, which enables a seamless, modular integration and conversion of any photovoltaic device to a PEC. The CAB is a bilayer that separates the desired functionalities into a barrier composed of a conductive, inert, and impermeable material such as graphite or titanium (here we used graphite) attached to the photovoltaic component using a conductive adhesive formulated with a polymer matrix and conductive fillers (carbons or metals). We first demonstrated greater than 99% translation of halide perovskite photovoltaic power to the chemical reaction in a standard three-electrode cell with individual PEC devices fabricated by depositing a catalyst-coated CAB on HaP photovoltaics (photocathode: p-i-n PSC|CAB|Pt catalyst, photoanode: n-i-p PSC|CAB|IrO_x_ catalyst). These measurements also revealed that our CAB is ambipolar, stably facilitating both reduction and oxidation reactions. We then performed unassisted water-splitting measurements on HaP-based photovoltaics using two different device designs. The first consisted of a CAB-protected photocathode and photoanode connected electrically in series, with optical illumination in parallel geometry, enabling unassisted operation with an STH efficiency of 13.4% with up to 16.3 h of operation to t_60_ and a total absorber geometric area of 0.24 cm^2^. We show that the stability of the integrated PEC in the serial case is limited by the n-i-p building block and not by the newly designed CAB. These results demonstrate a record efficiency for this serial PEC design based on HaP, but the practical efficiency of this co-planar architecture is limited to 15.5% due to the current density of the single-junction photovoltaic devices^13^. In contrast, HaP/Si tandems offer a promising low-cost photovoltaic system that has demonstrated a photovoltaic efficiency in the vicinity of 30%^17,32^. Therefore, to overcome the limitations of the serial co-planar system, we performed unassisted water-splitting measurements using a monolithic HaP/Si tandem photoanode by using an IrO_x_-coated CAB and Pt foil as the cathode. This system achieved an STH efficiency of 20.8% on a 0.44 cm^2^ area with 102 h of operation (t_60_), which to the best of our knowledge represents the state-of-the-art for integrated water-splitting PEC devices. More generally, we anticipate that the CAB and its combination with HaPs or any other earth-abundant photo-absorbers will serve as a widely applicable platform for driving other useful reactions and pave a path for developing low-cost technology for solar-to-fuel conversion.

### Highly efficient halide perovskite photovoltaic devices

**Fig. 1:**
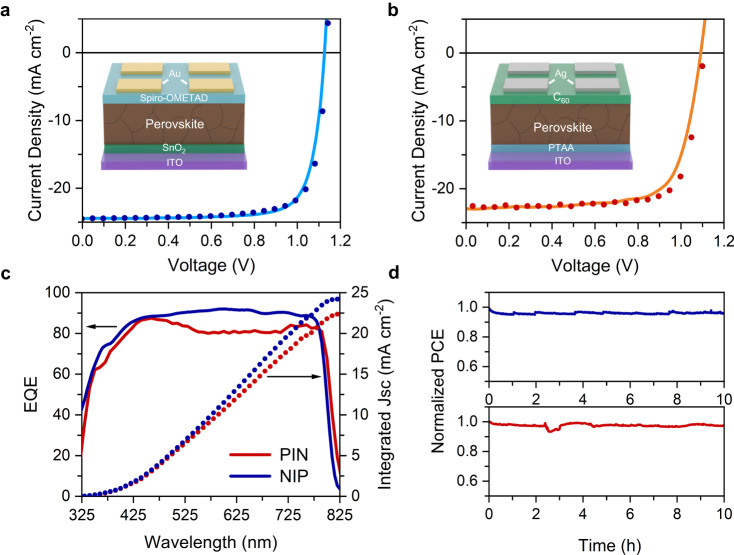
Photovoltaic performance and stability of perovskite solar cells. **a** Current-voltage curve for p-i-n (inverted) architecture PSC. **b** J-V curve for n-i-p (regular) PSC. **c** External quantum efficiency and integrated photocurrent of p-i-n (red) and n-i-p (blue) PSCs. **d** Stability of p-i-n (red) and n-i-p (blue) PVs over time with traditional epoxy encapsulation.

Figure 1 shows perovskite solar cell performance for both p-i-n and n-i-p (also known as inverted and regular, respectively) architectures as building blocks for the photocathode and photoanode, respectively. Figure 1a,b shows the current density-voltage (J-V) curves measured under AM 1.5G simulated sunlight with insets illustrating the corresponding device architecture. Perovskite solar cells typically possess a sandwich-like structure in which the active perovskite photo-absorber film is fabricated between charge-carrier-selective layers (hole transport layer and electron transport layer) for charge separation, with their respective metallic contacts for charge extraction. Transparent conductive oxides (indium-tin oxide ITO and fluorine-tin oxide FTO) act as charge collectors while allowing light to transmit with minimal parasitic absorption, while polymers (PTAA, C_60_, Spiro-OMeTAD) and metal oxides (SnO_2_) are the semiconducting charge-carrier transport layers used here. The p-i-n solar cell was formed by ITO/PTAA/Cs_0.05_FA_0.85_MA_0.1_Pb(I_0.95_Br_0.05_)_3_/LiF/C_60_/BCP/Ag layers to facilitate the transport of photogenerated electrons through C_60_ to the Ag electrode and holes through PTAA to the ITO (see Table 1 in “Methods” for material purity and details). The n-i-p stack was formed by FTO/SnO_2_/FA_0.97_MA_0.03_PbI_3_/Spiro-OMeTAD/Au such that the photogenerated holes pass through the Spiro-OMeTAD to arrive at the Au electrode and the electrons are collected by the FTO. The photovoltaic measurements revealed a power conversion efficiency (PCE) of 19.1% with short-circuit current of 22.8 mA/cm^2^, an open-circuit voltage of 1.10 V, and a fill factor of 0.76 from our champion p-i-n cells. Our best n-i-p cells yielded a PCE of 20.9% with short-circuit current of 24.4 mA/cm^2^, an open-circuit voltage of 1.13 V, and a fill factor of 0.76. Figure 1c shows the external quantum efficiency (or EQE) for the two cells. The integrated photocurrent density over the AM 1.5G spectrum of the n-i-p was measured to be 23.6 mA/cm^2^ and of the p-i-n 22.5 mA/cm^2^, which is within 5% of the short-circuit current density obtained from the J-V curves in Fig. 1a,b with differences arising from the slight spectral mismatch. We also measured the stability of the p-i-n and n-i-p solar cells under AM 1.5G illumination with maximum power point (MPP) tracking (ISOS L-1 protocol)^33^, which showed negligible degradation for 10 h.

### Barrier design for photoelectrodes

**Fig. 2:**
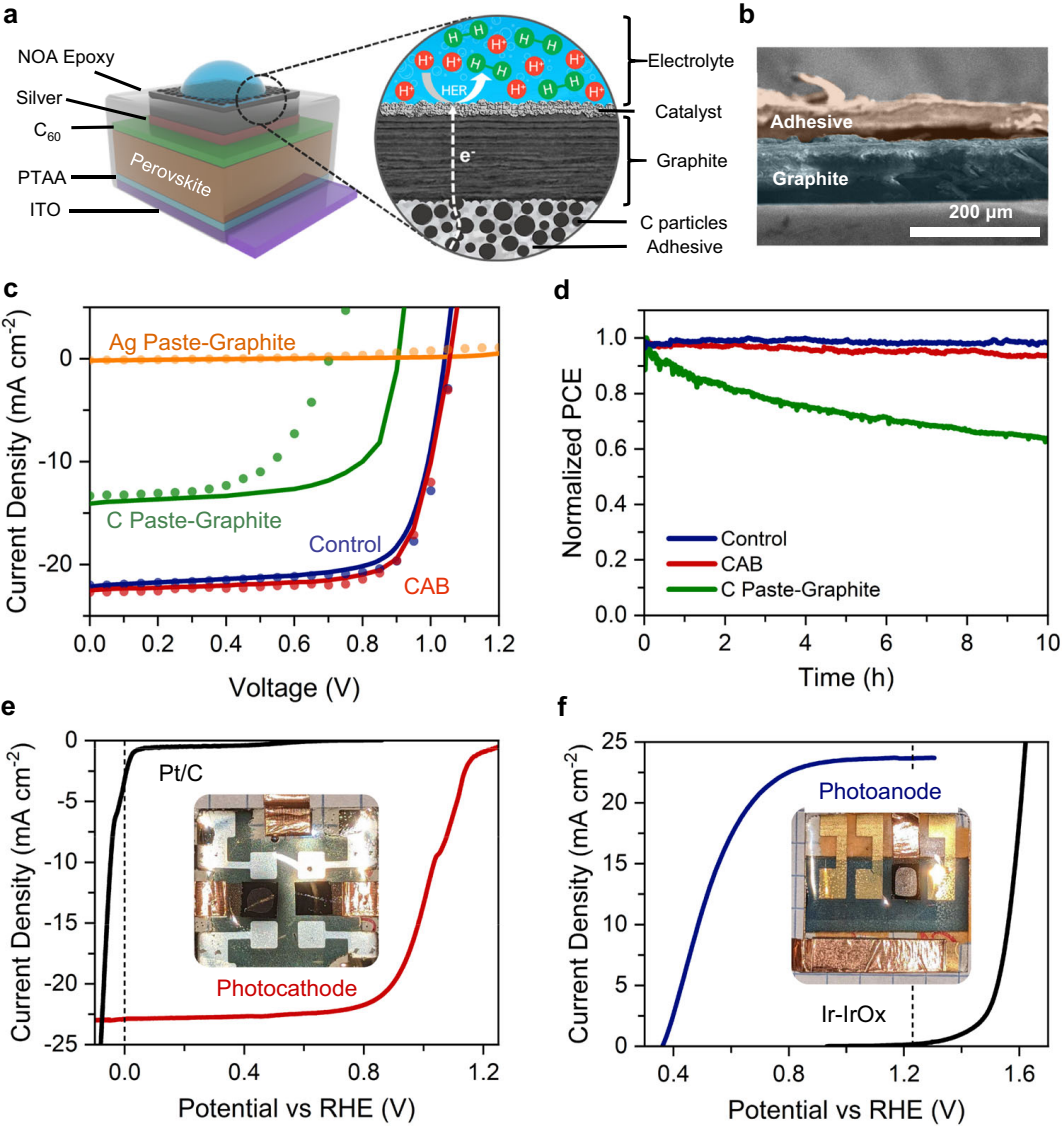
CAB (conductive adhesive-barrier) design for near-perfect preservation of photovoltaic efficiency. **a** Schematic design of a p-i-n perovskite solar cell transformed into a photocathode, with the circled region highlighted to depict the CAB|catalyst layers. The CAB|catalyst is attached to the top electrode of the PSC. **b** SEM image of CAB with graphite and C-based PSA. **c** Solar cell J-V measurements for devices with various methods of encapsulation, including glass-epoxy (control) and various commercial conductive adhesive inks capped with graphite. Lighter lines and smaller markers denote reverse sweeps. **d** Stability measurements of representative devices encapsulated with epoxy-glass (control), commercial C paste, and graphite barrier, and conducting C-filled PSA with graphite barrier (CAB). **e** Photocathode and **f** photoanode J-V measurements with electrocatalyst versus corresponding dark electrocatalyst-only in 0.5 M H_2_SO_4_ at 10 mV/s.

With the solar cells constructed, the crucial next step for the fabrication of the integrated PEC system was the development of the protective barrier for both photocathode and photoanode. This barrier must exhibit three key criteria: (1) low electrical resistance, (2) physical and chemical resistance to the ingress of corrosive electrolytes, and (3) compatibility with HaP surface termination (must not degrade any device layers already deposited). To initially evaluate the protective barriers against these design criteria, we screened multiple barrier materials using a simple qualitative color change stability test consisting of optical imaging of perovskite|barrier assemblies with small (~50 µL) drops of 0.5 M sulfuric acid deposited on top (Supplementary Fig. 1 and Supplementary Table 1) and measured the two-terminal electrical resistance by performing J-V scans (Supplementary Fig. 2). Our screening measurements implied that no single material adequately satisfied all the design requirements when deposited on perovskite solar cells. As a result, we developed a strategy to separate the desired functionalities into distinct materials whose combination satisfies all the design criteria. First, from our screening tests, we identified several viable barriers (Ti foil, Ni foil, Cu foil, graphite sheets of various origins and thicknesses), which prevent the ingress of the electrolytes and protect the underlying perovskite layer. Next, we developed an in-house composite polymer-conductive filler pressure-sensitive adhesive (PSA) that can adhere the barrier to the surface of the PSC without introducing solvent between the components^34,35^. The high conductivity of the PSA was achieved by blending the adhesive polymer with conductive particles (here, silver, copper, and amorphous carbon) to create conductive channels through the PSA to the barrier (see “Methods” for detailed synthesis and deposition steps). The tack, or stickiness, of the composite was tailored to achieve good physical adhesion without compromising on conductivity. Figure 2a illustrates the conductive adhesive-barrier (CAB) comprised of an amorphous C-blended PSA and a graphite barrier attached to the HaP electrode to convert it into a photocathode with the different components shown schematically in a magnified view. Figure 2b shows the corresponding SEM image of the CAB cross section. We measured the J-V performance of solar cells before and after attaching the CAB compared with systems using other conducting adhesive layers with graphite barrier added as shown in Fig. 2c. Our control solar cell showed a PCE of 17.7% and the addition of optimized CAB caused negligible changes to the PCE and other relevant figures of merit (V_OC_: 1.06/1.07, J_SC_: 22.0/22.7, FF: 0.76/0.73, for control and CAB, respectively). These changes may be ascribed to inter-sample variability rather than to a side interaction between HaP and CAB. In contrast, the commercial C paste-graphite dropped the PCE to about 5% with significant hysteresis and the Ag-paint degraded the HaP solar cell. We conducted continuous illumination MPP tracking for our barrier candidates and observe that our CAB retains 95% of the initial performance whereas the glass-epoxy encapsulated device (control) retained 98% and the C paste-graphite devices lost almost 40% of the initial performance in 10 h (Fig. 2d).

We note that the CAB offers a unique advantage compared to other strategies to fabricate barriers directly onto the photo-absorbers or photovoltaic surfaces. Here, the entire barrier and the catalyst deposition can be performed separately, in parallel, and adhered to the photovoltaic device using a dry transfer process at ambient temperature, pressure, and environment, thus mitigating the risk of degrading the perovskites due to leaving phases in commercial conductive inks (Supplementary Fig. 3). Because the design and deposition of the CAB directly address all three of the critical design criteria, there is substantial freedom in the selection of specific materials for the subcomponents. We demonstrated negligible changes to device J-V curves in the solid state for CAB-capped devices using alternative conductive fillers and barriers in Supplementary Fig. 4. Intuitively, the lack of material-specific dependence is unsurprising since the only design criteria probed in the solid state are compatibility with the perovskite surface termination, which is unchanged since we use the same method for deposition, and low electrical resistance. We anticipate that this flexibility in materials, which is a key feature of our approach, will enable photoelectrodes for other sensitive photo-absorbers beyond perovskites and for other electrochemical reactions and electrolyte compositions.

The capability of our CAB to create a robust physicochemical barrier with negligible loss in the photovoltaic performance enabled us to transform our high-efficiency photovoltaics into high-efficiency integrated PEC cells. Although the photovoltaic-CAB system is intrinsically capable of operation as a PEC in water, the catalytic activity of the graphite barrier alone for the hydrogen evolution reaction (HER) and oxygen evolution reaction (OER) was minimal (Supplementary Fig. 5). To achieve high catalytic activity for HER, we deposited a standard commercial Pt/C (20 wt%) nanoparticle catalyst at 0.5 mg/cm^2^ loading with Nafion binder^36^. The J-V curve of the Pt catalyst in 0.5 M H_2_SO_4_ is shown in Fig. 2e in black with a characteristically low overpotential at 20 mA/cm^2^ of 70 mV^37^. When deposited on the p-i-n HaP-PEC (with a CAB) and illuminated with AM 1.5G simulated sunlight, the onset potential shifted in the positive direction (anodically) by 1.1 V, matching the open-circuit voltage of the solar cell. The device achieved a photocathode efficiency, which we define as power contributed by the photo-absorber to the half-reaction beyond its standard potential, of 18.6% with a photocurrent of 21 mA/cm^2^ at 0.87 V vs. RHE (see Supplementary Note 1 and Supplementary Fig. 6 for a discussion of photoelectrode efficiency as a figure of merit, Supplementary Fig. 7 for photoelectrode power curves). We note that photoelectrode efficiencies do not translate directly to solar-to-hydrogen efficiencies, but they can be used diagnostically to identify losses associated with the degree of conversion of solar power into chemical, especially when compared with photovoltaic J-V curves. For OER, we loaded the CAB at 1 mg/cm^2^ with a synthesized nanoparticulate IrO_x_ catalyst ink suspension with a Nafion binder^38,39^. The J-V curve of the catalyst in 0.5 M H_2_SO_4_ in Fig. 2f in black shows an overpotential of 370 mV at 20 mA/cm^2^. When deposited on the photoanode similarly to the photocathode case and illuminated with 1-Sun simulated sunlight, the onset potential shifted negatively (cathodically) proportional to the open-circuit voltage of the solar cell. We calculated a photoanode efficiency of 11.3% with a maximum power photocurrent of 19.8 mA/cm^2^ at 0.66 V vs. RHE (Supplementary Fig. 7). As expected, the sluggish kinetics of OER resulted in a higher catalyst overpotential that consumed about 40% of the photovoltage at maximum power resulting in the lower photoanode efficiency. Because of the differences in reaction kinetics, the n-i-p solar cell’s larger PCE (20.9% vs. 19.1% in p-i-n) resulted in a lower photoanode efficiency (11.3% vs. 18.6% in photocathode). Nevertheless, these measurements clearly demonstrate that the CAB|Pt-electrolyte interface can almost perfectly translate the photovoltaic efficiency to drive the HER reaction due to low electronic resistivity and catalyst overpotential. We also show that this is not intrinsic to the specific materials used but to the CAB-catalyst design criteria in general by demonstrating a photocathode using a Ti barrier-based CAB and Pt/C catalyst for HER, which had 19.0% PCE in the solid state and a photocathode efficiency of 18.1% (Supplementary Fig. 8). These metrics represent a substantial quantitative leap in performance compared to prior photoelectrode-type devices using single-junction perovskites, with the highest half-cell efficiencies and the highest ratios between photoelectrode efficiency and parent solar cell PCE for both photocathode and photoanode (Supplementary Table 2).

### Co-planar series-connected tandem photocathode-photoanode

**Fig. 3:**
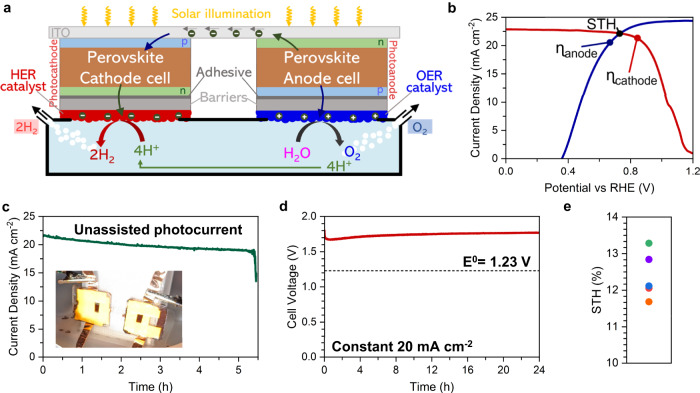
Series photocathode-photoanode unassisted water-splitting performance. **a** Schematic representation of the unassisted PEC water-splitting system using halide perovskite PECs. **b** Co-plotted photocathode and photoanode polarization curves from most efficient devices showing a maximum theoretical operating current density of 22.7 mA/cm^2^. **c** 2-electrode unassisted water-splitting stability test showing degradation after 5 h. **d** Electrocatalyst-only electrolyzer stability at 20 mA/cm^2^. **e** Distribution of STH efficiency across five devices, with an average STH of 12.4 ± 0.7%.

After demonstrating that the CAB can effectively translate the efficiency of a HaP photovoltaic to a PEC, we connected the photocathode and photoanode in series to drive an unassisted water-splitting reaction using the design illustrated in Fig. 3a. We designed a simple 3D-printed polypropylene semi-batch reactor with two openings in the sidewalls to expose the devices to the electrolyte, and gas inlet and outlet lines to pump inert gas and avoid pressurization in the reactor. We attached the CAB|catalyst layers to the p-i-n and n-i-p solar cell electrodes, encapsulated the surface surrounding the CAB with epoxy, and adhered the devices to the reactor sidewalls. After illuminating the integrated HaP-PEC with a 1-Sun AM 1.5G source, we measured the unassisted water-splitting current over time. The resulting water-splitting reaction is recorded with the visible onset of bubble formation with light illumination (Supplementary Movie 1). The intersection of the half-cell PEC J-V curves plotted with absolute current density gives the anticipated unassisted water-splitting current density for a 2-electrode system, which we measured to be about 22.1 mA/cm^2^ as illustrated in Fig. 3b. The initial photocurrent density in the 2-electrode measurement was in good agreement with this theoretical value at about 21.8 mA/cm^2^ (Fig. 3c). The Faradaic efficiency of the catalysts was measured to be unity (see Supplementary Fig. 9), as expected for Pt-based HER and Ir-based OER in 0.5 M H_2_SO_4_. Accounting for the 2x area contributions because of the two separate photo-absorbers (total area 0.24 cm^2^), the measured current density translated to a peak STH of 13.4%, calculated using Eq. 1. Our system with an STH of 13.4% and a continuous operation of 5 h is among the most efficient and stable for integrated PEC systems using halide perovskites and one of the first to achieve >10% STH, which is regarded as a technoeconomically relevant efficiency^31,40^. The device maintained an average current density of 20.1 mA/cm^2^ for 5 h with a gradual loss in current density (about 0.5 mA/cm^2^/h) before rapid failure. To understand the sudden degradation after 5 h, we independently measured the performance of the HER and OER catalysts in a standard electrolyzer geometry and confirmed that both catalyst materials were stable under constant-current control at 20 mA/cm^2^ for at least 24 h, with only a slight increase from 1.68 to 1.77 V cell voltage (Fig. 3d). To further understand the origin of the degradation for the integrated PEC system, we investigated representative post-reaction solar cell J-V curves from devices used in unassisted water-splitting, which reveal significantly degraded n-i-p devices and largely intact p-i-n devices (Supplementary Fig. 10). Finally, we measured the time-dependent photocurrent of a representative photocathode at 0 V vs. RHE for 60 h and found minimal degradation, although we note that this only highlights the protective ability of the barrier and does not demonstrate stable power delivery or correlate to unassisted water-splitting stability (Supplementary Fig. 11). These findings highlight the robust ability of the CAB against corrosion for long time periods and indicate that the origin of the degradation is predominantly from the loss in efficiency of the n-i-p solar cells. We performed 5 experiments on similar PECs and found consistent peak STH (Fig. 3e) but some differences in stability (t_60_ from peak photocurrent) from 2 to 16.3 h (Supplementary Fig. 12). We note that, unlike for the PSC stability measurements conducted in Fig. 1d, the deposition of CABs and encapsulation with epoxy occurred in atmospheric air, which may significantly accelerate the degradation rate of the Spiro-OMeTAD based hole transport layer in the n-i-p building block.

### Monolithic silicon-perovskite tandem photoanode

Single-junction devices like those used for the co-planar system have a fundamental efficiency limit  due to the parallel orientation of the junctions. The practical STH efficiency limit using state-of-the-art catalysts is around 15.5%^13^. Our measured efficiency is primarily lower due to a mismatch in the maximum power points of our PECs as shown in Fig. 3b; however, it is not straightforward to further improve the efficiency of our solar cells or reduce the overpotential of our catalysts. Therefore, we changed the photo-absorber architecture to the monolithic stacked tandem, which can generate adequate photovoltage to split water with only one photoelectrode. Stacked tandems can practically achieve STH efficiencies up to 22.8% (although the detailed balance limit, assuming small catalyst overpotentials, is much higher at 33%)^13,41^. We chose to use monolithic silicon-perovskite tandem solar cells, which have achieved more than 31% PCE^17^, to demonstrate the versatility of our CAB in terms of compatibility with diverse photovoltaic elements. Furthermore, the sensitive HaP layer in this architecture is the wide-gap absorber using p-i-n architecture and the Ag-terminated silicon interface is on the electrolyte side, mitigating stability losses observed in our 1-junction HaP photoanodes.

**Fig. 4:**
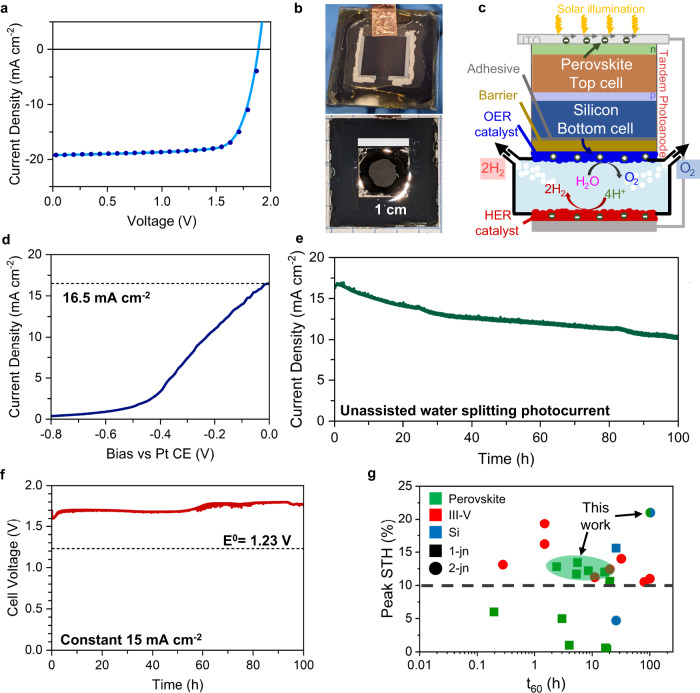
Tandem silicon-perovskite photoanode unassisted water-splitting performance. **a** Schematic representation of the unassisted PEC water-splitting system using HaP/Si tandem. **b** Optical images of absorber (top) and catalytic (bottom) faces of transformed silicon-perovskite photoanode. **c** HaP/Si tandem photovoltaic J-V curve. Scale bars are 1 cm. **d** 2-electrode J-V curve of photoanode from champion device showing a theoretical operating current density of 16.9 mA/cm^2^. **e** 2-electrode unassisted water-splitting over time. **f** IrO_x_ nanoparticulate and Pt foil electrocatalyst 2-electrode time-dependent voltage at 15 mA/cm^2^. **g** Comparison among other integrated PEC-type devices with respect to STH and lifetime at t60. Demonstrations not reaching t_60_ are shown with full reported lifetime.

We fabricated a 1.1 cm^2^ silicon-halide perovskite (Si/HaP) tandem solar cell based on the work of Albrecht et al.^32^ with an initial PCE ~29% (Supplementary Fig. 13). The device EQE with integrated photocurrent is shown in Supplementary Fig. 14. This tandem device was stored for 3 months (in the dark, under inert atmosphere) before being integrated into the PEC device, and right before deposition of the CAB|catalyst it still yielded a solar cell efficiency of 27.7% as illustrated in the J-V curve in Fig. 4a. The tandem exhibited an open-circuit voltage of 1.90 V, a short-circuit current density of 19.2 mA/cm^2^ and a fill factor of 0.76 under AM 1.5G illumination. We transformed the tandem photovoltaic cell into a photoanode by attaching the Ir/IrO_x_ coated CAB to the Si face of the tandem. We encapsulated the non-active area of the tandem on the Si face with epoxy and covered the HaP face with a sheet of glass that was sealed with epoxy along the edges (Fig. 4b). We then adhered the device to a 3D-printed polypropylene reactor, in the design schematically shown in Fig. 4c. Figure 4d shows the J-V characteristics of the tandem photoanode in 2-electrode mode with a Pt foil counter electrode (CE), yielding an observed current density of 16.5 mA/cm^2^ at zero bias vs. Pt CE. The measured current density was slightly lower than that measured at the maximum power point of the photovoltaic (1.61 V and 17.2 mA/cm²), which was consistent with the overpotential losses of our OER and HER catalysts. We then allowed the device to operate continuously at short-circuit condition with AM 1.5G 1-Sun illumination for 102 h and monitored the current density as shown in Fig. 4e. We measured a peak photocurrent of about 16.9 mA/cm^2^ after 1 h of illumination, which corresponds to an STH efficiency of 20.8% using unity Faradaic efficiency. In light of the high metrics achieved, we further confirmed that the silicon-perovskite photoanode-Pt cathode system furnishes unity Faradaic efficiency using online gas chromatography (Supplementary Fig. 15).

Over the course of 102 h of continuous operation, the photocurrent degraded to its t_60_ value of about 10 mA/cm^2^, corresponding to an STH efficiency of 12.3%, after which we turned off the solar simulator. To understand the origins of the degradation, we conducted separate measurements in a two-terminal electrolyzer geometry on the IrO_x_ and Pt foil catalyst system at a constant current of 15 mA/cm^2^ as shown in Fig. 4f. At a current density of 15 mA/cm^2^, which was close to the peak current demand of the unassisted photoanode (in Fig. 4e) we found that the required cell potential increased from 1.6 to 1.8 V, indicating that the observed loss of photoanode efficiency over time is partially due to catalyst degradation. We conducted surface SEM characterization on IrOx-graphite substrates before and after water-splitting measurements and observed that large sections of IrOx delaminated from the graphite (Supplementary Fig. 16). XPS measurements also showed the appearance of new peaks corresponding to oxidized C species after the reaction, suggesting that some activity loss may be ascribed to the barrier conductivity at the surface or contact resistance (Supplementary Fig. 17). In addition, the tandem devices used here utilized a LiF interlayer at the electron selective contact, which was shown to reduce the PCE of unencapsulated tandem solar cells to 90% of the initial PCE under constant maximum power point tracking for 100 h^32^. These factors imply that degradation originates in both the photovoltaic and the catalysts but is more significant in the catalysts.

Figure 4g shows a comparison between reported STH efficiency and lifetime at t_60_ for notable non-concentrator integrated solar water-splitting devices across several materials classes in the past few decades including those demonstrated here^1,2,8–10,15,30,31,42–49^ (an alternative version using lifetime at t_90_ is shown in Supplementary Fig. 18 and a more detailed listing including efficiencies from half-cell only demonstrations using halide perovskite photo-absorbers is in Supplementary Table 3). Most devices above 10% STH (dashed line in Fig. 4g) are based on multijunction III-V semiconductors. Our integrated series photocathode-photoanode (Fig. 3c) is the most efficient single junction HaP-based system to date and with a high lifetime. Our HaP/Si PEC is to the best of our knowledge the first non-concentrator PEC to exceed 20% STH, regardless of material or number of junctions, and with a relatively high t_60_ and an active area of 0.44 cm^2^. These results represent a critical step forward for building integrated high efficiency PEC systems using low-cost photo-absorbers with high efficiency. The CAB platform is highly versatile and can be applied to a wide variety of photo-absorbers and combined with a broad suite of catalysts to photoelectrochemically power arbitrary redox reactions. Furthermore, its method of synthesis and deposition is promising for scaled device fabrication. This technology provides a path to a rich research domain with critical opportunities for solar fuel devices with solar concentration, modularization, and device scaleup. Future studies should focus on the fabrication of highly efficient perovskite-based photoelectrodes, the incorporation of lower-cost catalysts through improved precious metal utilization or incorporation of non-PM catalysts, and reactor designs for improved overall performance and economics.

## Methods

All chemicals were used as received, as stated in Table 1, unless otherwise stated.

**Table 1:** Materials and purity

Chemical	Purity	Cat No.	Supplier
DMF	99.8%	227056	Sigma-Aldrich
DMSO	99.9%	276855	Sigma-Aldrich
Diethyl ether	>98%	676845	Sigma-Aldrich
Chlorobenzene	99.8%	AC396970010	Acros
Acetone	99.9%	A949-4	Fisher Chemical
Ethanol	>99.2%	2701	Decon
FTO	N/A	FTO-P001	Kaivo Optoelectronics
ITO	N/A	ITO- P001	Kaivo Optoelectronics
PTAA	N/A	702471	Sigma-Aldrich
PbI_2_	99.99%	L0279	TCI Chemicals
PbBr_2_	99.999%	398853	Sigma-Aldrich
CsI	99.999%	203033	Sigma-Aldrich
MAI	99.99%	MS101000	Greatcell Solar
FAI	99.99%	MS150000	Greatcell Solar
Spiro-OMeTAD	>99%	HT0728	One material
C_60_	99.5%	379646	Sigma-Aldrich
BCP	96%	140910	Sigma-Aldrich
Ag (pellets)	99.99%	EVMAG40EXEB	Kurt J. Lesker
Au (pellets)	99.99%	EVMAUXX40G	Kurt J. Lesker
Pt on graphitized carbon	20 wt%	738549	Sigma-Aldrich
IrCl_3_ · *x* H_2_O	99.9%	203491	Sigma- Aldrich
Hexadecyltrimethylammonium bromide	>99%	H9151	BioXtra
NaBH_4_	≥98.0%	452882	Sigma-Aldrich
Nafion 117	~5%	70160	Sigma-Aldrich
Polypropylene filament	N/A	N/A	Dynamism
Polyurethane	N/A	N/A	The Gorilla Glue Company
H_2_SO_4_	72%wt	LC25645	LabChem
Epoxy	N/A	NOA68	Norland Products
Isoamyl acrylate (stabilized with HQ)	>98.0%	A089025ML	FischerSci
Acrylic acid (stabilized with Hydroquinone Monomethyl Ether)		8001810500	Sigma-Aldrich
Ethyl acetate	>99.5%	319902	Sigma-Aldrich
Heptane	>99%	H2198	Sigma-Aldrich
Silver NPs (<150 nm particle size)	99%	484059	Sigma-Aldrich
Pyrolytic graphite sheet		EYGS182303	Panasonic/Digi-Key

### Perovskite solar cell device fabrication

#### Architecture p-i-n

##### ITO/PTAA/Cs_0.05_FA_0.85_MA_0.1_Pb(I_0.95_Br_0.05_)_3_/LiF/C_60_/BCP/Ag

For the p-i-n perovskite devices, the device structure was ITO/PTAA/Cs_0.05_FA_0.85_MA_0.1_Pb(I_0.95_Br_0.05_)_3_ /LiF/C60/BCP/Ag^50^. The glass/ITO substrates were treated with UV ozone for 15 min before use. The hole transport layer of PTAA was made by spin-coating a 1.5 mg/mL toluene solution at 5000 RPM for 30 s and then annealed at 100 °C for 10 min. The perovskite films were fabricated by anti-solvent approach in a N_2_ glovebox. The perovskite precursor (1.5 M) was prepared by mixing CsI, FAI, MABr, PbBr_2_, PbI_2_ chemicals in a stoichiometric ratio, and dissolved in a mixed solvent of DMF:DMSO (4:1 v/v). The two-step spin-coating procedure with 1000 RPM for 10 s and 4000 RPM for 40 s was used. 150 µL chlorobenzene was dropped onto the spinning substrates during the 20 s of the second spin-coating step, followed by 100 °C annealing for 10 min. Finally, C_60_ (30 nm), BCP (6 nm), and Ag (100 nm) in sequential order were thermally evaporated under high vacuum.

##### Architecture n-i-p: FTO/SnO_2_/(FAPbI_3_)_0.97_(MAPbI_3_)_0.03_/ Spiro-OMeTAD/Au

The n-i-p PSCs were fabricated with the device architecture FTO/SnO_2_/(FAPbI_3_)_0.97_(MAPbI_3_)_0.03_/ Spiro-OMeTAD/ Au. The SnO_2_ layer was deposited on FTO using the chemical bath deposition (CBD) method^51^. After SnO_2_/FTO substrates were treated with UV ozone for 15 min, perovskite precursor was spin-coated on the substrate at 500 rpm for 10 s, 1000 rpm for 10 s, and 5000 rpm 15 s, and 1 mL of ethyl ether was dripped onto the substrate being spin-coated. The perovskite layer was annealed at 150 °C for 15 min in an ambient environment. The perovskite precursor was prepared by mixing 1.55 M of FAI, 1.55 M of PbI_2_, and 0.05 M of MAPbBr_3_ with 35 mol% of methylammonium hydrochloride in a mixed solvent of DMF and DMSO (8:1 v/v). The spiro-OMeTAD solution was spin-coated at 4000 rpm for 20 s. The spiro-OMeTAD solution was prepared by mixing 90 mg/mL of spiro-OMeTAD in chlorobenzene with 23 μL of lithium bis(trifluoro-methanesulfonyl) imide salt (540 mg/ml in acetonitrile), 10 μL of FK209 salt (375 mg/mL in acetonitrile) and 39 μL of 4-tert-butylpyridine. Finally, a 100-nm-thick gold electrode was deposited by thermal evaporation.

### Perovskite solar cell characterization

The solar cell current-voltage characteristics were measured using a Keithley 2400 Source meter at a rate of 0.05 V/s from 0 to 1.2 V (Forward scan) and 1.2 to 0 V (Reverse Scan). AM 1.5G (100 mW/cm^2^) illumination was provided by 450 W Xe lamp solar simulator (Oriel LCS 100). The simulated solar illumination intensity was calibrated with a Newport Si reference cell.

### Photovoltaic EQE

The external quantum efficiency was measured using a QUANTX300 EQE system from 320 to 820 nm wavelength with a step of 5 nm and a chopper frequency of 40 Hz.

### Photovoltaic stability

The solar cells were encapsulated in UV-cured epoxy (Norland Optical Adhesive 68) and the stability was measured using maximum power point (MPP) tracking under simulated AM 1.5G (100 mW/cm^2^) using the above solar simulator.

### Pressure-sensitive adhesive synthesis

To create a pressure-sensitive adhesive (PSA), compatible monomers are copolymerized according to classical methods^34,35^. A representative protocol follows: For a batch, the following species were added to a reactor under an inert (argon/nitrogen) atmosphere: 3.55 g ethyl acetate + 3.55 g isooctyl acrylate + 0.05 g methacrylic acid. A stock solution of the initiator was created by mixing 0.005 g of benzoyl peroxide with 3 g of ethyl acetate. The stock solution was stirred for 5 min before 1 g of the stock solution was added to the reaction vessel. The reaction vessel was maintained at ~55 °C and stirred continuously. After 90 min the following components were added to the reaction vessel: 0.02 g methacrylic acid + 1 g of the initiator stock solution. After 5 h, 1 g of the initiator stock solution was added. After about 10 h, the reaction was deemed complete, and the product was diluted to a desired viscosity via the addition of heptane (between 1 and 10 mL heptane added per 0.1 g of polymer product). The PSA was made conductive via the addition of conductive carbon or silver nanoparticles to create electrically conductive channels (e.g., 40 mg/mL diluted polymer solution). An optimized batch of conductive PSA has a weight loading of 97.5 wt.% heptane, 2 wt.% PSA, and 0.5 wt.% carbon nanoparticles. Three-hundred microliters of this solution could be utilized to coat a 1-in^2^ anticorrosion barrier (graphite) via spin-coating at 1000 RPM for 1 min.

### CAB fabrication

Graphite sheets were coated on one face with highly conductive PSA via spin or spray coating, then placed on a hotplate at 100 °C for 10 min to remove the leftover solvent. The resulting CAB was flipped to have the sticky side down and heated to 60 °C for catalyst ink drop-casting. The ink was allowed to dry completely to create the final CAB-catalyst structure.

### 1-junction PEC fabrication

Unencapsulated solar cell devices were screened in atmospheric air for activity with J-V curves as described above. Completed CAB catalysts were then carefully placed over the top contacts of the solar cells and pressed down gently. The copper tape was used to create leads to the transparent conductive oxide (bottom contact) and to the top contact of the devices for independent photovoltaic performance testing. The surface of the device except for the area protected by the CAB was then sealed with epoxy (NOA68) and cured in air under UV light (UVP UVL-21, Analytik Jena US) for 25 min at approximately a one-inch distance. The resulting catalyst geometric area, defined by the bounds of the epoxy, was measured using a high-resolution camera and calculated using MATLAB’s Image Processing Toolbox, to ensure a close match with the photo-absorber geometric area. Finally, the completed photoanode/photoanode was adhered to the reactor using polyurethane and allowed to dry in atmospheric air for 8 h before use. The measured photocurrents were normalized to the photo-absorber area which was defined by an aperture.

### Silicon-perovskite PEC fabrication

The copper tape was used to create a lead to the metallic contact on the perovskite face, after which a glass slide was placed on the perovskite face and the edges were sealed with epoxy. Completed CAB-IrOx catalysts were carefully placed over the Ag contact on the silicon face of the device and gently pressed to achieve good adhesion. The area around the CAB catalyst was then encapsulated with epoxy which was cured in an Ar-filled glovebox (>99.5%) under UV light for 2 min. The resulting catalyst geometric area was then measured and used to define an equal aperture area. Finally, the completed device was adhered to the reactor using polyurethane and allowed to dry in atmospheric air for 8 h before use.

### Iridium electrocatalyst synthesis

The iridium-based catalyst synthesis was based upon recent work from Lettenmeier et al. on the development of nano-sized IrO_x_-Ir catalysts for proton exchange membranes^38,39^. Broadly, the catalyst particles were prepared through the chemical reduction of a surfactant/iridium salt mixture in an O_2_-free environment. In this work, particle synthesis was achieved by retrofitting a Friedrichs condenser (ChemGlass 330 mm, 24/40 Joint) as a jacketed reactor. The condenser’s liquid jacket was cooled with a recirculating water bath to maintain a temperature of 15 °C throughout the synthesis. Nitrogen gas (Airgas, 99.9%) was introduced through the upper hose connection which sparged through the inner drip tube to both provide agitation and prevent the introduction of atmospheric oxygen into the system. The lower hose connection led to a beaded pipe-to-hose glass adapter attached to the bottom of a Claisen adapter. The upper port of the adapter serves as an exhaust port to prevent pressure build-up within the condenser while the lower port houses a 125-mL separatory funnel which was used to charge the condenser with the reactant solutions.

In a typical synthesis, the salt and surfactant solutions were first prepared in separate vials: 88.2 mg of iridium chloride hydrate (Sigma-Aldrich IrCl_3_ · *x*H_2_O, 99.9% trace metal basis) was dissolved in 50 mL absolute ethanol (Decon) while 1.31 g of hexadecyltrimethylammonium bromide (Sigma-Aldrich CTAB, BioXtra ≥99%) was dissolved in 40 mL of absolute ethanol. The iridium solution was first poured into the separatory funnel and then charged into the reactor with the nitrogen flow rate at approximately 300 cm^3^ min^−1^. Once complete, the CTAB solution was fed through an identical manner. An additional 20 mL of absolute ethanol was then introduced into the funnel to ensure a complete transfer of the reactants into the condenser. Once charged, the flow rate was increased to 500 cm^3^ min^−1^ and left to agitate/equilibrate for 90–120 mins. At this time the chemical reductant was prepared by dissolving ~114 mg sodium borohydride (Sigma-Aldrich NaBH_4_ powder, ≥98.0%) in 50 mL of ice-cold ethanol. This solution was then immediately poured into the separatory funnel and the N_2_ flow rate was reduced to 400 cm^3^ min^−1^. The reductant was then dripped into the CTAB/Ir solution at a rate of approximately 2.5 mL min^−1^. Once all the NaBH_4_ solution was transferred the sparging rate was again increased to 500 cm^3^ min^−1^ and left for 3 h.

Post reaction, the contents of the condenser was divided into several 50 mL centrifuge tubes, and the solids were isolated at 5000 rpm (6000×*g*) for 5 mins. After decanting, absolute ethanol was used to combine the solids into a single tube which was subsequently filled to volume and sonicated (~5 mins) to completely resuspend the solids. This solution was again centrifuged at 5000 rpm (6000×*g*) for 5 mins. This process was conducted two additional times, with a 50/50 mixture of deionized water and ethanol and then just DI water, to thoroughly remove excess surfactant and/or chloride. Finally, the solids were left to dry under vacuum (−25 psig) at 60 °C overnight (12–16 h) before being weighed.

Iridium inks were prepared by simply suspending the as-prepared catalysts in absolute ethanol (1 mg/mL) with the addition of 10 µL of Nafion 117 (Sigma-Aldrich ~5% in a mixture of lower aliphatic alcohols and water) and sonicating this mixture for at least 15 mins prior to drop-casting the ink.

### Electrochemical measurements

All electrochemical measurements were performed on a Bio-logic VSP potentiostat using 3D-printed single-compartment electrochemical cells. In all 3-electrode measurements, Ag/AgCl (sat. KCl) was used as the reference electrode; however, all potentials shown herein have been converted to RHE using the Nernst equation:

$${V}_{{RHE}}={V}_{{meas}}+{V}_{{RE}}^{0}+0.059\times {pH}$$

where V_RHE_ is the potential versus RHE, V_meas_ is the measured potential from the potentiostat, and V_RE_^0^ is the reduction potential of the reference electrode. Reference electrodes were calibrated by a master Ag/AgCl (sat. KCl) reference electrode which was only ever exposed to saturated KCl electrolyte.

The counter electrode, unless otherwise specified, was a graphite rod. The electrolyte for acidic measurements was 0.5 M H_2_SO_4_, and all pH’s were tested using a calibrated pH probe to be within the range of 0.3–0.4 (Thermo Scientific Orion Star A111 with VWR Reference Standard Buffers).

All electrochemical measurements were obtained from electrolytes that were degassed with Ar flow for 10 mins at >20 sccm and constantly exposed to 20 sccm Ar during measurement. The sweep rate for voltammograms was 10 mV/s from open-circuit potential. A magnetic stir bar was used to remove bubbles. During stability tests (chronopotentiometry, chronoamperometry), the fresh electrocatalyst was swept from open-circuit potential to the stability setting (a current density for CP or a voltage for CA) and held thereafter while the corresponding parameter (voltage for CP or current density for CA) was measured.

During bias-free water-splitting measurements, for the co-planar system, a photocathode and photoanode were connected in series with the potentiostat with no reference electrode (2-electrode mode). For the monolithic tandem photoanode, the photoanode was connected to the WE cables and the Pt foil to the CE cables. In both cases, the RE cable was electronically connected to the counter electrode. The devices were exposed to AM 1.5G simulated sunlight and the current was measured with no electrical bias.

### Faradaic efficiency measurements

Faradaic efficiency measurements were performed on samples composed of adhesive, barrier, and electrocatalyst only and on silicon-perovskite tandem photoanodes. The assemblies were encapsulated with epoxy in air as described above and mechanically pressed to a flow-based two-compartment electrochemical reactor separated by a Nafion membrane. The inlet flow was 0.5 M H_2_SO_4_ which was pre-bubbled and stored in a headspace with inert gas (no O_2_). For the catalyst-only measurements, the catalysts were operated in 2-electrode mode using a Pt-based cathode for measurements of our OER catalyst or an IrO_x_ mesh for measurements of our HER catalyst. The catalysts were held at a constant current of approximately 20 mA/cm^2^, with slight variation (~1%) due to instrument control, and the products were quantified using an online gas chromatograph. For the silicon-perovskite photoanode, the anode was replaced by the photoanode and the cathode was an area-matched Pt/C-coated CAB prepared as described, and the system was held at short-circuit under illumination. Products were again quantified using an online gas chromatograph.

### 3D-printed polypropylene reactor

There are no standard designs for photoelectrochemical reactors. We chose to design a tank reactor for simple 3-electrode PEC measurements which we have modified for 2-electrode and bias-free measurements and gas collection. In the 3-electrode case, we used a cube shape with rounded internal edges to facilitate flow and minimize contamination between experiments. The reactor had a square base with a side length 3.8 cm, 4.0 cm height, and 0.2 cm wall thickness. Two holes were also added along the center top side of the reactor with a diameter of 0.8 and 1.0 cm to insert working and reference electrodes into the electrolyte. Two protruding cylinders with an internal diameter of 0.125 inches were included on the top corners for the inert carrier gas inlet and product gas outlet. To minimize losses from incident sunlight, we decoupled the interfaces for light absorption and electrocatalysis. The reactor was designed with a hole centered on a side wall (1.0 cm diameter), using the wall as an adhesion surface to create a seal to which the PEC was adhered.

Printing an enclosed reactor is difficult due to the top “ceiling.” A dual extruder capable of switching between polypropylene and a dissolvable support material was necessary to build a foundation for the top layers of the reactor. In this case, PVA (polyvinyl alcohol) was the ideal support material due to its low cost and solubility in water. Unfortunately, the smooth surface of polypropylene (PP) makes adhesion of the first layer of PVA difficult. If not layered correctly, PVA easily compacts on the extruder nozzle which can cause expensive damage and a ruined print. An alternative is a detachable lid which can be printed separately and additionally allows facile cleaning of the reactor. Threaded twist caps with a fitted O-ring offer a compromise between print complexity and tight sealing, and represent the best opportunity for an easily reusable design, while the enclosed print remains the best option for potential product gas removal.

The PEC reactor body required a chemically inert material to prevent degradation due to the presence of extreme pH and high ionic strength media with extended use. 3D printing with PP filament offered a cheap, inert body that created a watertight seal when layered. To adhere PP coming out of the extrusion nozzle at 428 °F to the 3D printer bed, we increased the bed temperature to 212 °F (from 150 °F) which stopped the rapid cooling and resolved layer misalignment. A brim added extra material to the first layer to broaden the base of the print and reduce permeability. Both changes contributed to reducing the impact of contractive forces after cooling and provided the following layers with a solid base.

For the printing process, we used an Ultimaker 3 with dual extruders of nozzle size 0.4 mm that produced a 0.48 mm line width when laid on the print bed. With these specifications, the layer height was 0.2 mm after material adhesion with a recommended flow rate of 50 mm/s and extruded at 428 °F. Recommended heuristics indicated that the extrusion flow rate should stay between 90 and 110% to reduce the chance of layer misalignment. The Ultimaker 3 comes programmed with pre-determined settings for printer bed temperature, extruder temperature, and infill density for all compatible filaments, and unless otherwise specified default settings were used.

## Supplementary information

Supplementary Information

Description of Additional Supplementary Files

Supplementary Movie 1

## Data Availability

All data are available in the main text or the supplementary materials.
