# Supplementary material for: Integrated halide perovskite photoelectrochemical cells with solar-driven water-splitting efficiency of 20.8%
Source: Nat Commun. 2023 Jun 26;14:3797. doi: 10.1038/s41467-023-39290-y (PMC10293190; doi:10.1038/s41467-023-39290-y)
Supplement: Supplementary file 1 — Supplementary Information [file 41467_2023_39290_MOESM1_ESM.pdf]

Supplementary Information for

Integrated halide perovskite photoelectrochemical cells with solar-driven water-splitting efficiency of 20.8%

Austin M.K. Fehr, Ayush Agrawal, Faiz Mandani, Christian L. Conrad, Qi Jiang, So Yeon Park, Olivia Alley, Bor Li, Siraj Sidhik, Isaac Metcalf, Christopher Botello, James Young, Jacky Even, Jean Christophe Blancon, Todd G. Deutsch, Kai Zhu, Steve Albrecht, Francesca M. Toma, Michael Wong*, Aditya D. Mohite*

Correspondence to: adm4@rice.edu & mswong@rice.edu

This file includes:

Supplementary Text Note 1
Supplementary Figs. 1 to 18
Supplementary Tables 1 to 3

Supplementary Text

Supplementary Note 1 – Modeling half-cell efficiency

$$\boldsymbol{\eta_{photoelectrode}} = \frac{J_{op} \times |E_{op} - E_0|}{P_{sunlight}}$$

A photovoltaic J-V curve (**Supplementary Fig. 5A**) allows calculation of the maximum power point of a solar cell (starred) by multiplying voltage and current density at every point and selecting the largest resulting quantity. A similar equation exists for the calculation of maximum power in a photoelectrode (**Supplementary Fig. 5B**, in yellow), but it is necessary to account for lost power (**Supplementary Fig. 5B**, in blue) to catalyst overpotential by using the standard reduction potential of the half-reaction under study as a new zero. It is important to note that the photoelectrode efficiency is necessarily lower than the photovoltaic efficiency due to catalyst overpotential. One can roughly quantify losses due to added anticorrosion barriers by summing the power lost to overpotential and the power supplied to the reaction at the current density at the MPP, and comparing to the parent photovoltaic. When the difference between these numbers approaches zero, the anticorrosion barrier has not degraded the device or added series resistance.

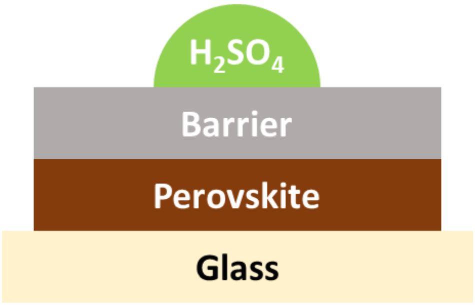

Supplementary Fig. 1. Drop Test (Color Change Stability Screening). We performed high throughput screening of various barrier materials by depositing them on top of a Glass|Perovskite surface, dropping 20-50uL of 0.5M H_2SO_4 on the barrier and observing the color change of the perovskite film underneath. Potential candidates were eliminated if the perovskite film degraded in the presence of electrolyte (0.5 M H_2SO_4), which was readily visible to the naked eye due to the characteristic and distinct color changes associated with water-mediated perovskite degradation.

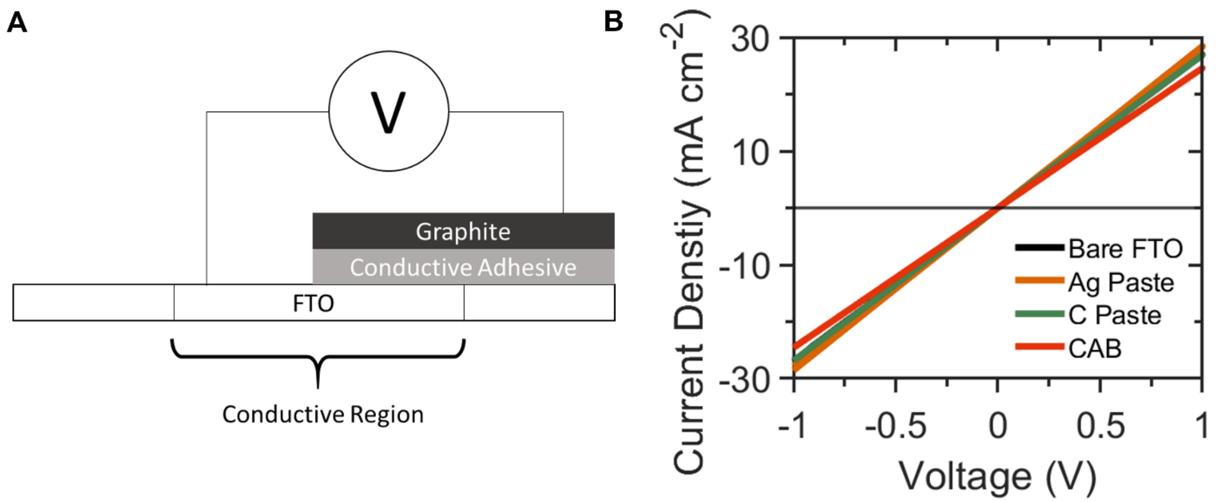

Supplementary Fig. 2. Resistor curves of CABs with varying adhesives. A. Schematic illustrating the setup to measure the resistance of various adhesives for the barrier. **B.** J-V scans of Ag paste, C paste and inhouse synthesized CAB. All the investigated conductive adhesives have similar resistance curves, relative to the bare FTO control. The conductive PSA (CAB) has the added advantage of not requiring a destructive curing stage when adhering the anti-corrosion barrier to the photovoltaic.

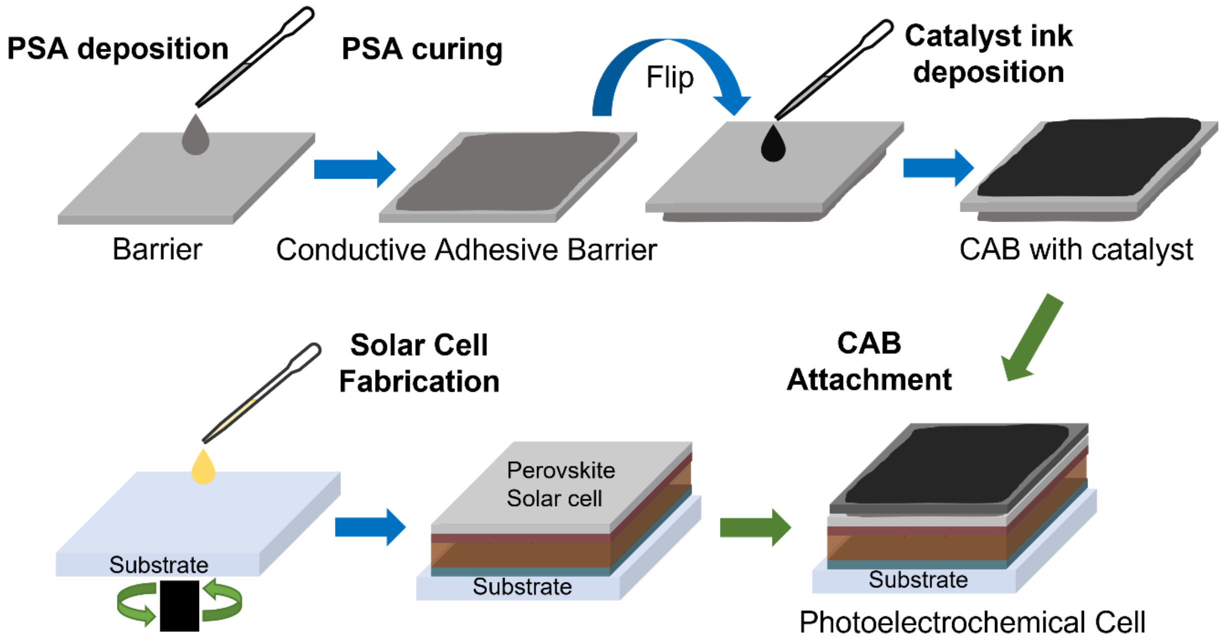

Supplementary Fig. 3. Workflow for depositing CAB with dry transfer. This is a schematic describing the creation of catalyst coated CABs and their application to solar cells to form photoelectrodes. This technique decouples solar cell fabrication from CAB fabrication enabling the use of solvents and processing conditions that would be detrimental in sequential layer-by-layer fabrication traditionally used for photoelectrode devices.

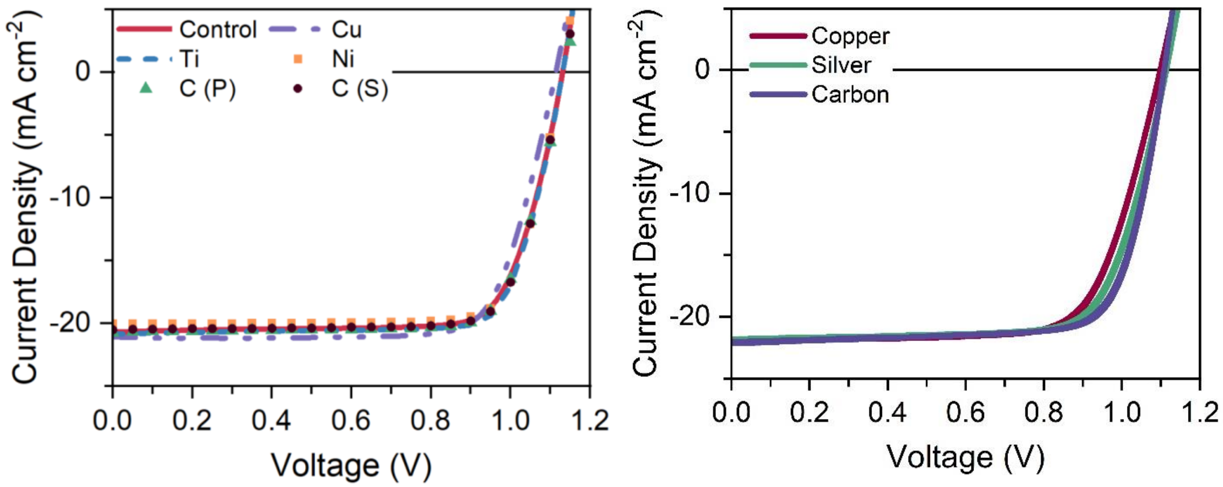

Supplementary Fig. 4. The effect of alternative fillers and alternative barriers on solid-state solar cell J-V curves. A. J-V curves for p-i-n perovskite solar cells with various barrier materials in CAB, including Cu (copper), Ti (titanium), Ni (nickel), C(P) (carbon from Panasonic), C(S) (carbon from Sigma). **B.** J-V curves for p-i-n perovskite solar cells with various adhesive filler materials.

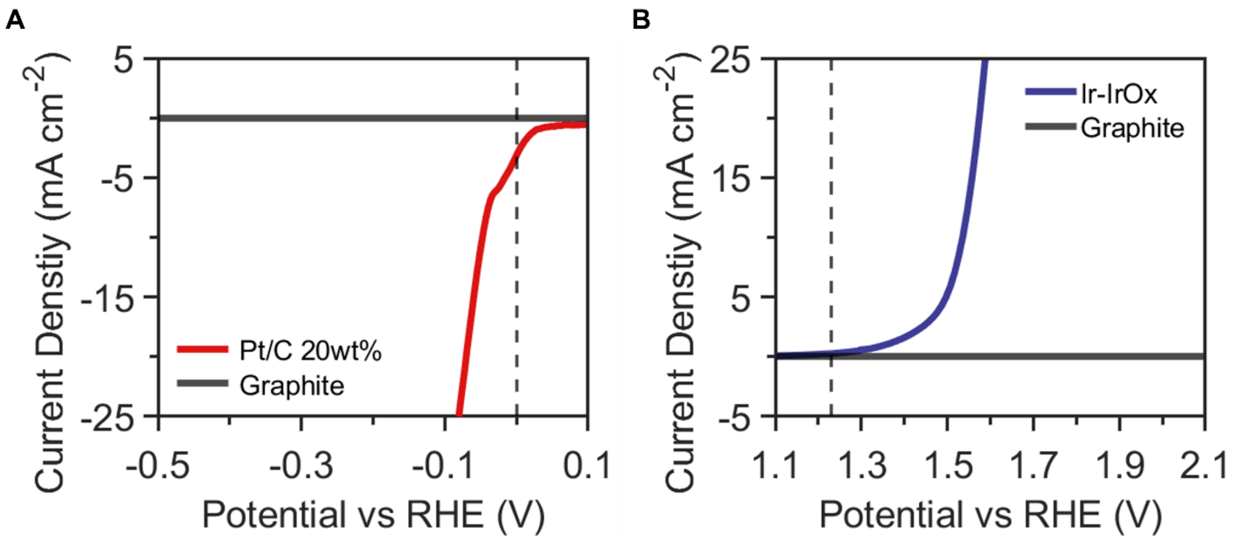

Supplementary Fig. 5. Electrocatalyst activity before and after depositing catalysts on CAB. **A.** HER catalyst activities on graphite and Pt/C on graphite. **B.** OER catalyst activities on graphite and Ir-IrOx on graphite. Bare graphite exhibits negligible activity in the range of potentials observed (-0.5 to +2.1 V vs RHE in 0.5 M H_2SO_4).

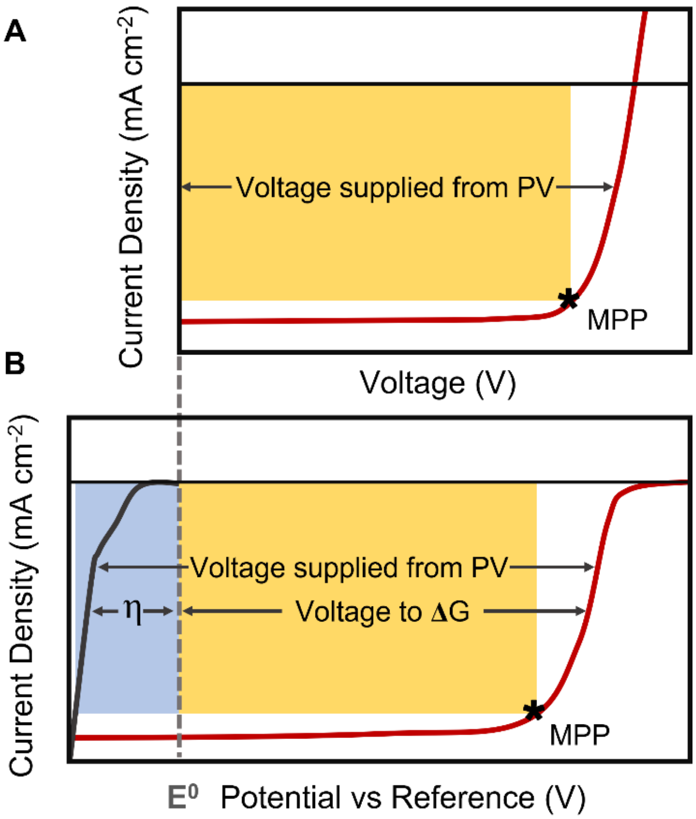

Supplementary Fig. 6. Photovoltaic and Photoelectrode Efficiencies. A. Model photovoltaic solid-state illuminated J-V curve. **B.** Model photoelectrode and dark electrocatalyst J-V curve in 3-electrode mode.

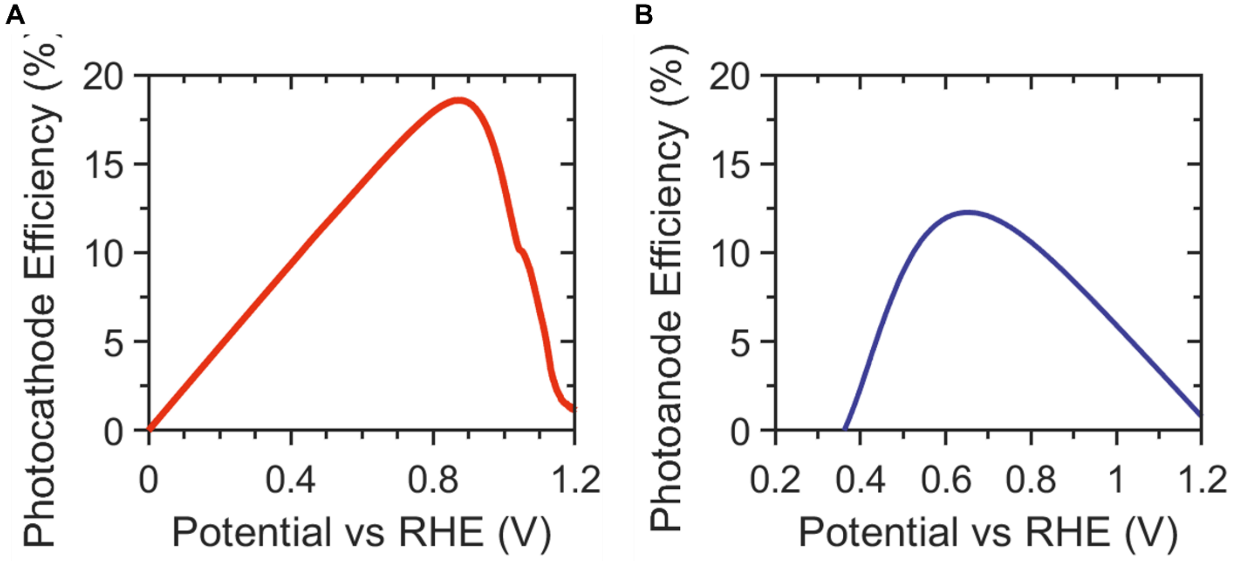

Supplementary Fig. 7. Photoelectrode Power Curves for A. Photocathode, **B.** Photoanode used in the Fig. 2e and 2f of the main text. Photoelectrode efficiency was determined via SI Discussion 1. The photocathode was created via combination of a p-i-n perovskite solar cell with a CAB coated with 0.5 mg/cm^2 of 20wt% Pt/C commercial catalyst with Nafion binder. The photoanode was created via combination of an n-i-p perovskite solar cell with a CAB coated with Ir-IrOx nanoparticles at a 1mg/cm^2 loading with Nafion binder. The LSV was conducted with respect to an Ag/AgCl reference electrode with a graphite rod serving as the counter electrode in 0.5 M H_2SO_4.

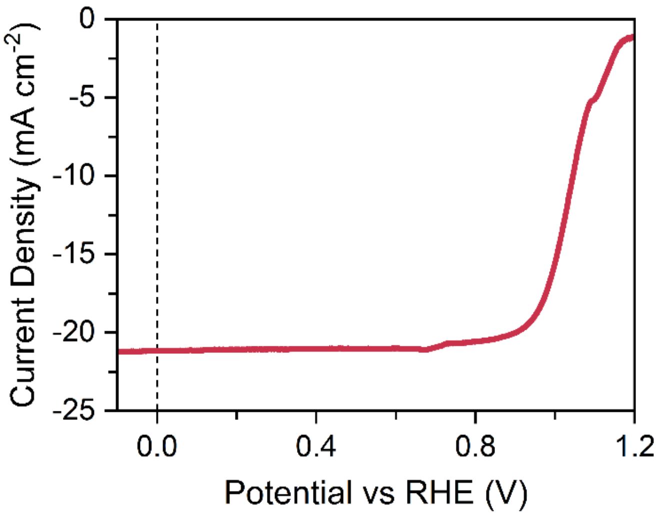

Supplementary Fig. 8. Performance of Ti-barrier photocathode. J-V curve showing photocathode efficiency of 18.1% compared to a PV efficiency of 19% (Supplementary Fig. 4).

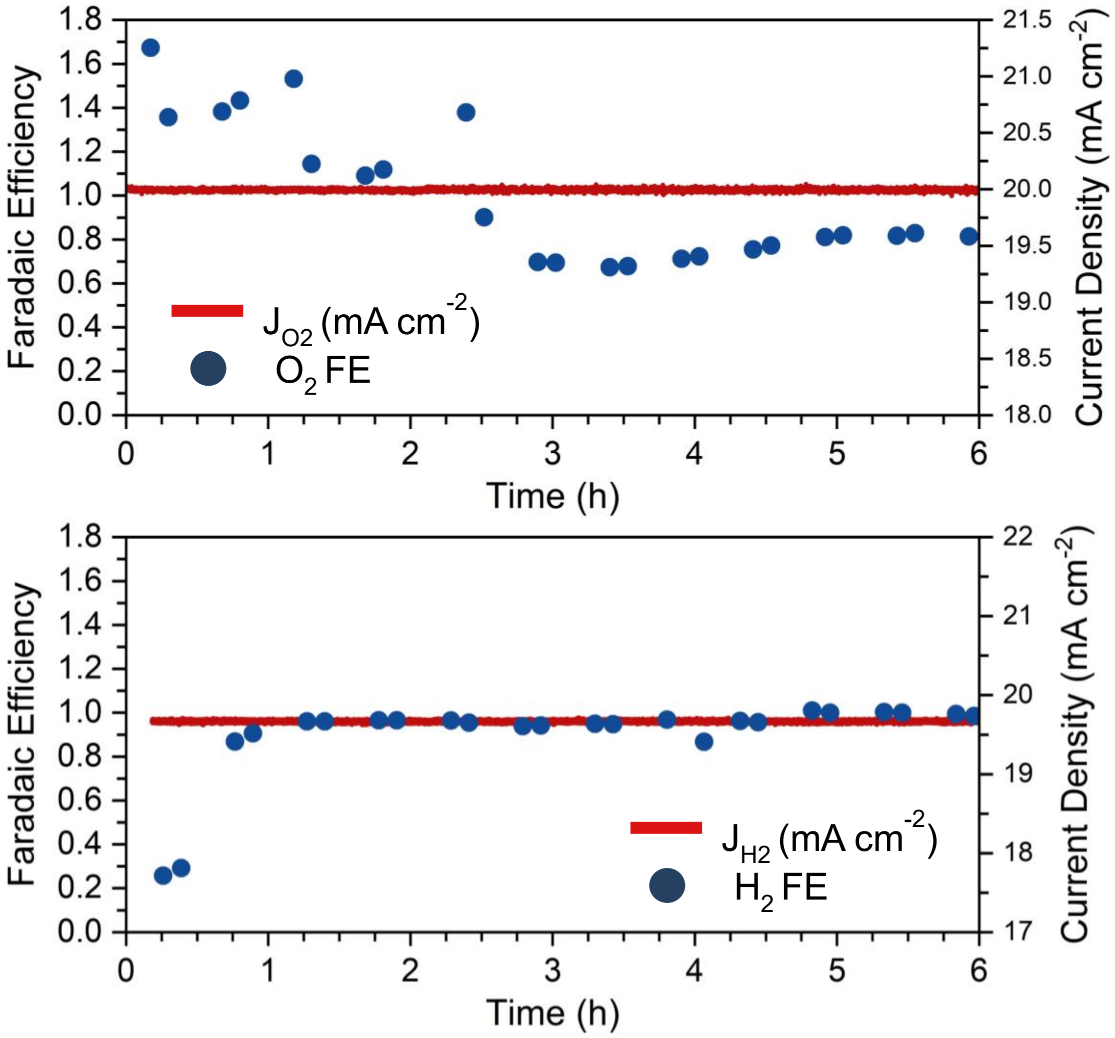

Supplementary Fig. 9. Faradaic Efficiency for the representative catalysts. A. HER for which the Pt/C, 20 wt%, 0.5mg/cm^2 achieved an average Faradaic efficiency to H_2 of 98% +/- 2%, which we have taken as unity in our calculations of efficiency, and **B.** OER for which the Ir-IrO_x, 1 mg/cm^2 achieved an average Faradaic Efficiency for O_2 between 1 and 6 hours of 89% +/- 25%.

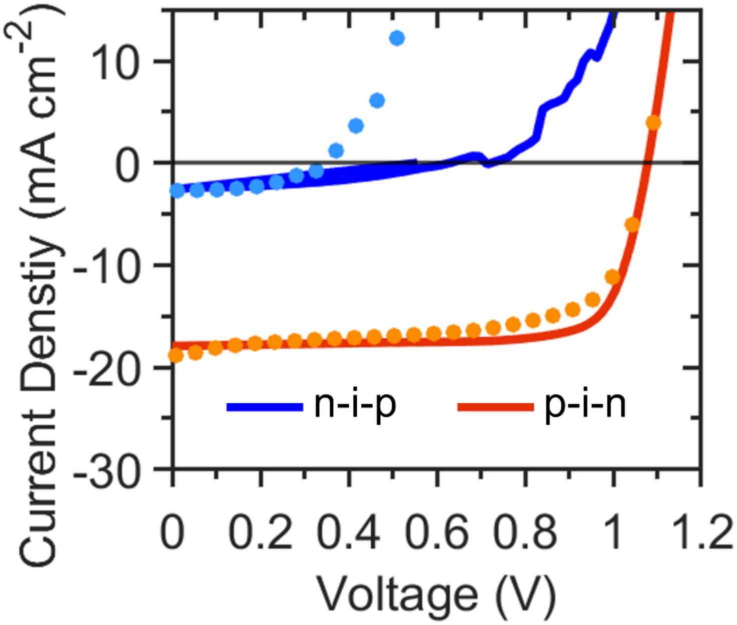

Supplementary Fig. 10. Representative post-reaction photovoltaic J-V curves. Representative solid-state J-V curves photoelectrodes after 18h of bias-free water splitting reaction show a degraded n-i-p device and largely stable p-i-n device. Solid lines forward sweep, dotted lines reverse.

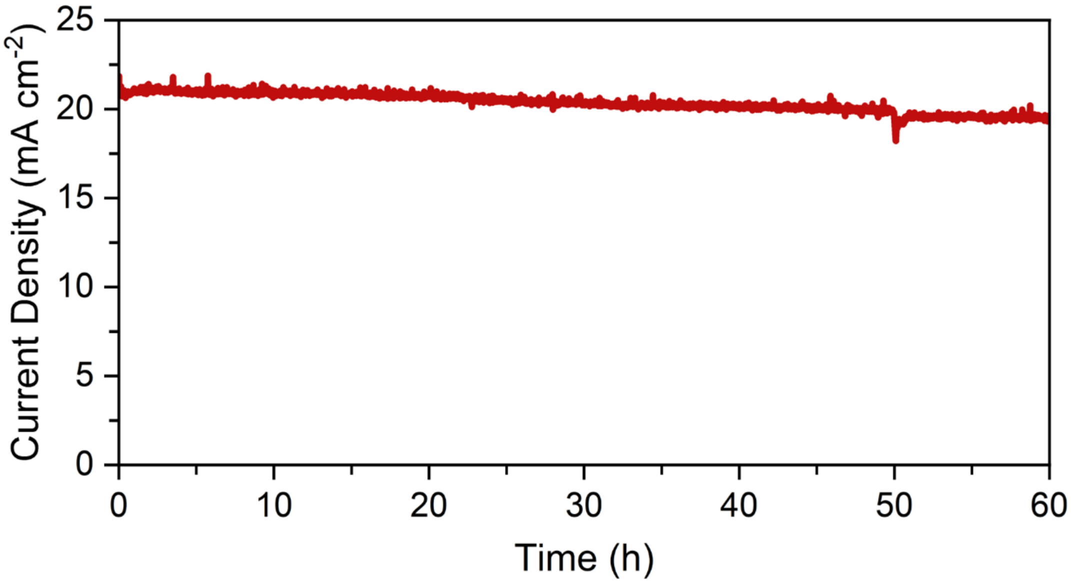

Supplementary Fig. 11. Representative chronoamperometric trace of photocathode at 0V vs RHE. Representative J-t curve for photocathode held at 0 V vs RHE. Since the overpotential of the Pt catalyst is low and the semiconductor is held near short-circuit, it is not reflective of the stability of the photocathode during unassisted water-splitting. These data are included only to demonstrate that the barrier is a robust against to chemical diffusion-mediated degradation of the device.

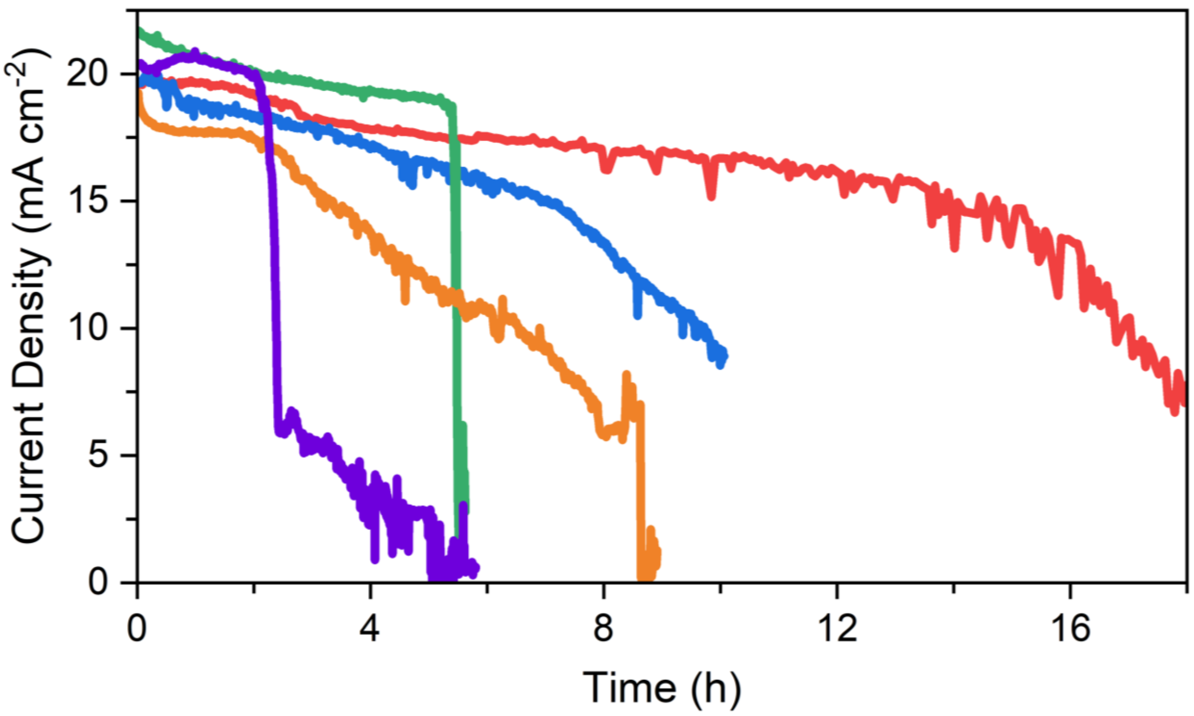

Supplementary Fig. 12. Variation of stability for 5 representative devices. Current density vs Time plots for the 5 best co-planar serial water splitting devices. The initial current density is well-clustered. The mean peak photocurrent was 20.2 mA cm^{-2} with a standard deviation of 1.1 mA cm^{-2}. The mean t_{60} was 7.6 hours with a standard deviation of 5.6 hours. The mean t_{90} was 3.5 hours with a standard deviation of 1.5 hours. The large variation in stability is a result of inter sample variability and changes in processing conditions, particularly relative humidity in atmosphere and exposure time, affecting the photoanode devices.

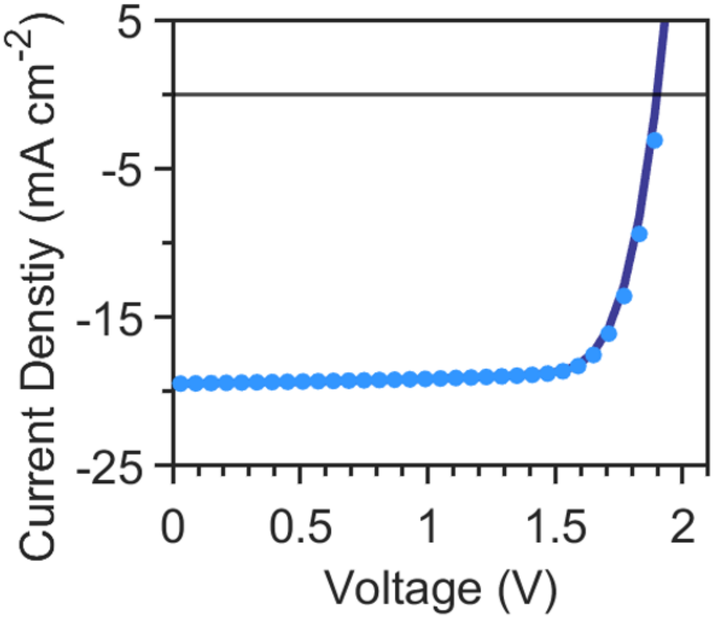

Supplementary Fig. 13. Initial silicon-perovskite tandem photovoltaic J-V curve. The initial PCE of the Si-Perovskite tandem photovoltaic was 29% with a short circuit current of 19.5 mA cm^{-2}, open circuit voltage of 1.91V and a Fill Factor of 0.78.

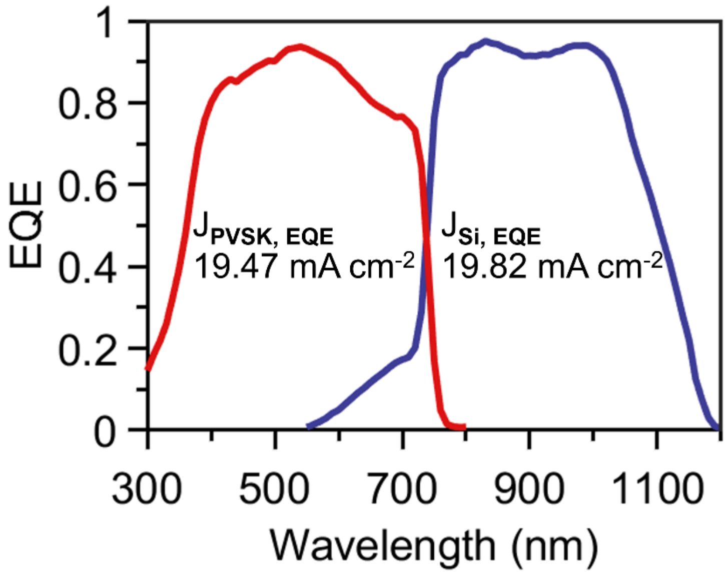

Supplementary Fig. 14. Silicon perovskite tandem photovoltaic EQE characteristics. External quantum efficiency of the silicon-perovskite tandem used for the bias-free water-splitting PEC.

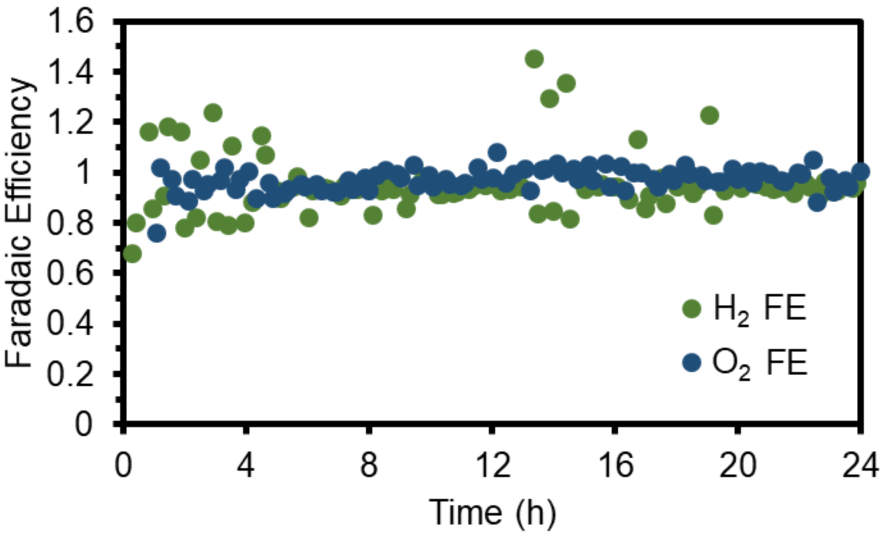

Supplementary Fig. 15: Silicon-perovskite tandem photoanode-Pt cathode online gas analysis. Online gas chromatography of hydrogen and oxygen show Faradaic efficiencies within error of unity. The average FE of H_2 was 95.6% +/- 12.1% and the average FE of O_2 was 97.3% +/- 4.3%.

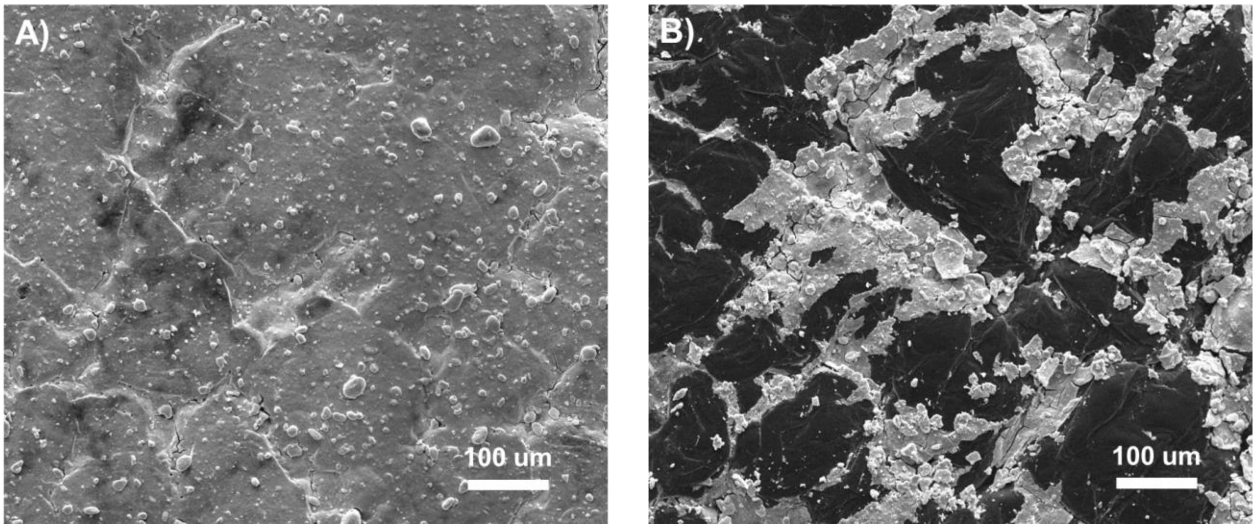

Supplementary Fig. 16: Surface SEM images of IrO_x-graphite A) before and B) after a 24h continuous 2-electrode water splitting electrolysis done at a current density of 20 mA cm^{-2}.

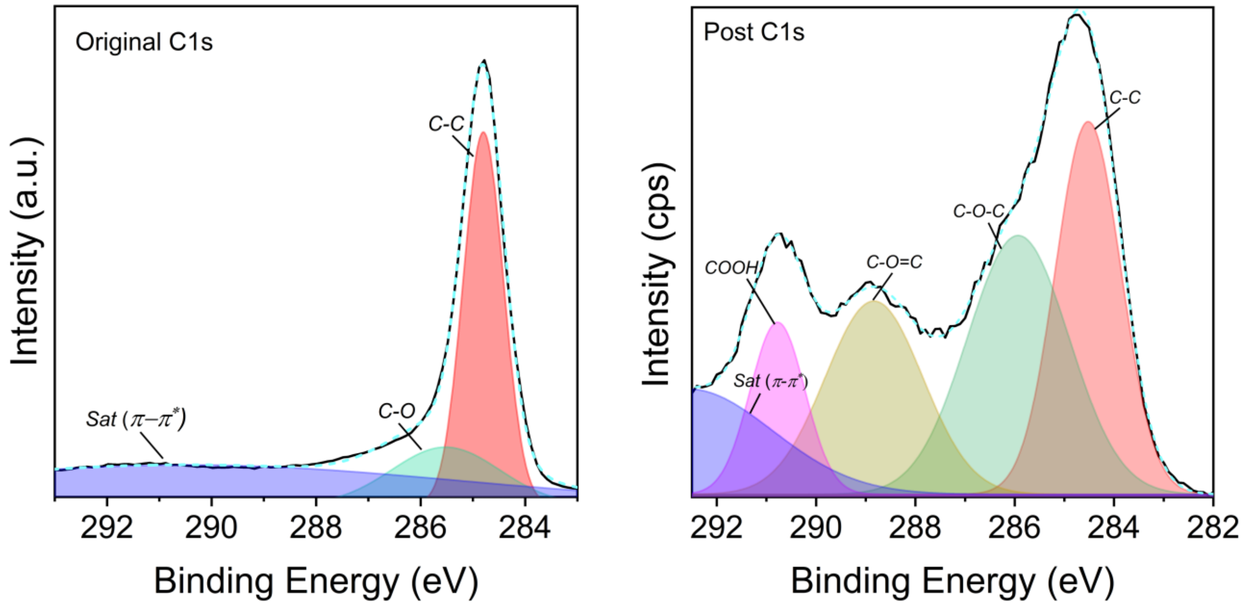

Supplementary Fig. 17: XPS measurements on IrO_x-graphite surface before and after 2-electrode constant current OER measurements.

5

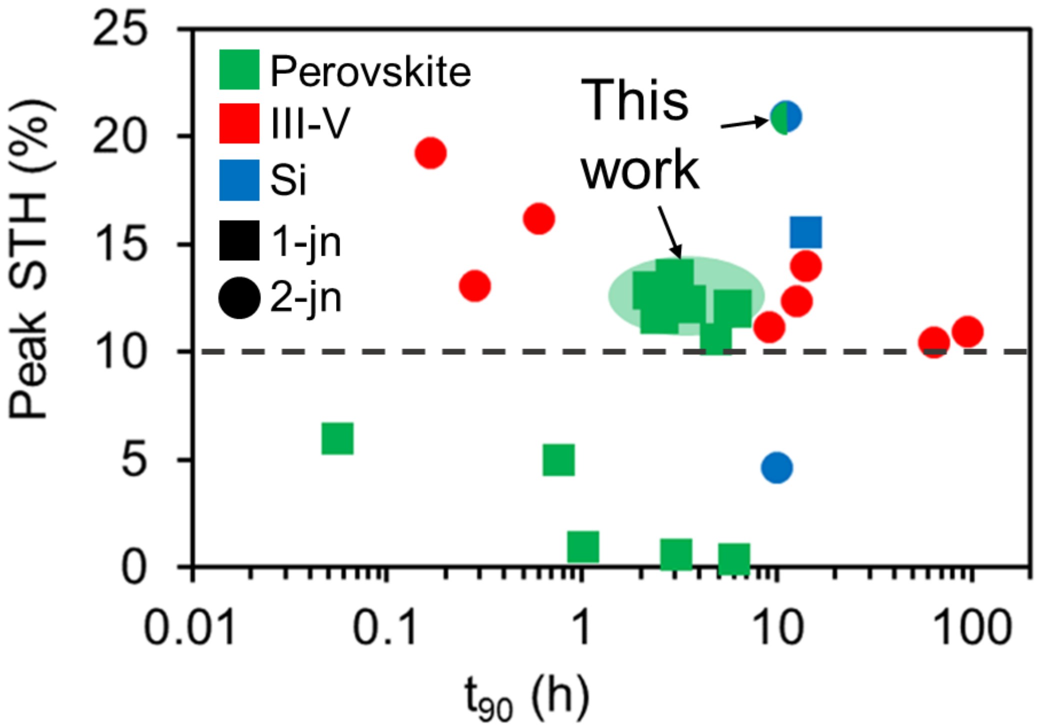

Supplementary Fig. 18. PEC comparison plot featuring reported peak STH and lifetime at t_{90}. Lifetimes were read from reported graphs taking the best unassisted water-splitting stability.

Supplementary Table 1. High performing anti-corrosion barriers performance under 0.5 M H_2SO_4. Various anti-corrosion materials, barrier thicknesses, and deposition techniques with their rationale are presented. Images of the sample prior to the start of the drop test and upon optical observation of the perovskite film degradation are presented, with observations regarding the rate of degradation and noticeable changes in the film quality. (**WCA**: Water contact angle)

Material	Reasoning	Before	After	Observation
**Pt film**<br>50nm<br>Sputtered	Inert metal, conductive, catalytic	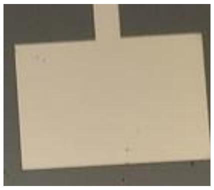	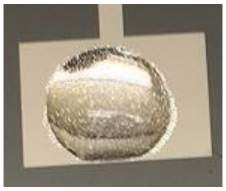	Dendritic growths originating with roughness from perovskite, coupled with yellowing of perovskite layer
**Oriented carbon nanotubes (CNTs)**<br>50nm<br>Wet-transferred with chlorobenzene	Dense, ordered CNTs hinders water diffusion, maintains conductivity, and solvent for transfer is compatible with perovskite	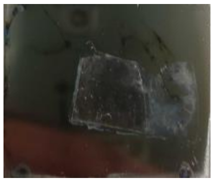	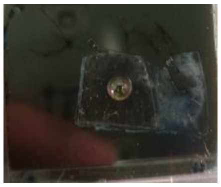	Perovskite and barrier damaged during transfer, macroscopic holes in CNTs film cause immediate barrier failure and electrolyte permeation
**PMMA film**<br>30-300nm<br>Spin-coated	Thick enough PMMA can be highly water-resistant and blended with conducting material (percolation)	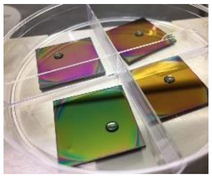	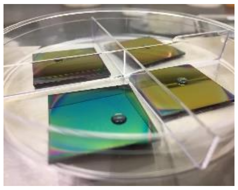	Pure PMMA at >100nm thickness can protect perovskite from color change for >24h, but WCA does change and below 50nm thickness results in significant yellowing
**Graphite sheet**<br>30-100um<br>Attached	Highly conductive and thick enough to be impermeable to water, also stable at various pH	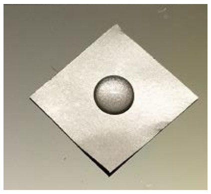	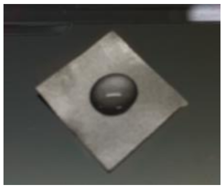	>24h with no morphology loss, minimal change to WCA

Supplementary Table 2. Comparison of efficiencies in solid state and as photoelectrodes among perovskite single-junction devices.

Ref	Electrode	PCE	Barrier	Catalyst	PE	PE/PCE
[21]	Cathode	11.4	FM	Pt	2.0	0.18
[24]	Cathode	11.1	C+Epoxy	Pt	5.0	0.45
[23]	Cathode	20.3	GaIn+Ti	Pt	10.0	0.49
[25]	Cathode	15.5	CCP+Ni	Ni-CoP	10.1	0.65
**This work**	**Cathode**	**19.1**	**CAB**	**Pt**	**18.6**	**0.97**
[26]	Anode	15.6	FM	Ni	4.3	0.28
[27]	Anode	16.0	Graphite	Ni	5.0	0.31
[22]	Anode	4.1	Graphite	Ir	1.5	0.37
[25]	Anode	20.4	CCP+Ni	NiFe	8.9	0.44
**This work**	**Anode**	**20.9**	**CAB**	**Ir**	**11.3**	**0.54**

Supplementary Table 3. Photoelectrochemical device comparison papers, including some reports of halide perovskite-based half cells and general unassisted water-splitting demonstrations.

Architecture	Ref	STH	t_{60} (h)	t_{90} (h)	$\eta_{cathode}$	η_{anode}
Si-PVSK|CAB|Catalyst	This work	20.8%	102	11		
n-i-p + p-i-n PSC|CAB|Catalyst		13.4%	16.3	6	18.6%	11.3%
n-i-p + p-i-n PSC|CCP|Ni|Catalyst	2	10.6%	20	5	10.1%	8.9%
$BiVO_4$|Ti|PET|PSC|epoxy+C|Pt	3	0.6%	17	3	5%	
PSC|FM|Ti-Foil|Catalyst + $BiVO_4$|TiCo	4	1%	4	1		
2 PSC|Surlyn|Glass|FTO|Catalyst	5	6%	0.2	0.1		
2 PSC|PIB|Coverglass|Catalyst	6	5%	3	0.8		
PSC|FM|Catalyst+ $BiVO_4$|TiCo	7	0.4%	18	2.5		
PSC|EGaIn|Ti-foil|MoS_2	8				11%	
PSC|FM|Pt	7				8%	
PSC|EGaIn|Ti-Foil|Pt	9				10%	
PSC|AZO|FM|Catalyst	10				5%	
PSC|(PCBM+ALD TiO_2)|Catalyst	11				6%	
PSC|Ag-paint|Ti-Foil|Catalyst	12				8%	
PSC|FM|Catalyst	13				3%	
PSC|Graphite|Ni	14					6%
PSC|CCP|Ni-foil|NiFe	15					9%
PSC|Au-Ni	16					2%
Perovskite|m-carbon|Graphite|Catalyst	17					1%
Perovskite|CC|Ag-paint|CC	18					1%
PSC|FM|Catalyst	19					3%
PSC|Ni	20					1%
Catalyst|SS|3Jn-Si|ITO|Catalyst	21	4.7%	26	10		
3 BBJ-Si|Catalyst	22	15.6%	26	14		
GaAs|InGaP|TiO_2|Catalyst	23	10.5%	80	63		
GaAs|InGaP|TiO_2|Catalyst	24	11%	100	95		
Ge|GaInAs|GaInP|AlInP|Catalyst	25	14%	32	14		
Catalyst|SSplate|Ge|GaAs|InGaP|TiO_2|Catalyst	26	11.2%	11	9		
GaInAs|GaInP|Catalyst	27	16.2%	1.5	0.6		
‘p-on-n’ + ‘n-on-p’ GaAs|Ti|Catalyst	28	13.1%	0.3	0.3		
Catalyst|GaAs|GaInAs|GaInP|AlInP|TiO_2|Catalyst	29	19.3%	1.5	0.2		
$GaInP_2$|nGaAs|pGaAs|Catalyst	30	12.4%	20	12.5		

5

1. Holm, R. *Electric Contacts*. *Electric Contacts* (1967). doi:10.1007/978-3-662-06688-1_4
2. Rhee, R. *et al.* Unassisted overall water splitting with a solar-to-hydrogen efficiency of over 10% by coupled lead halide perovskite photoelectrodes. *Carbon Energy* 1–10 (2022). doi:10.1002/cey2.232
3. Andrei, V. *et al.* Floating perovskite-BiVO4 devices for scalable solar fuel production. *Nature* **608**, 518–522 (2022).
4. Edwardes Moore, E., Andrei, V., Zacarias, S., Pereira, I. A. C. & Reisner, E. Integration of a Hydrogenase in a Lead Halide Perovskite Photoelectrode for Tandem Solar Water Splitting. *ACS Energy Lett.* **5**, 232–237 (2020).
5. Liang, J. *et al.* A Low-Cost and High-Efficiency Integrated Device toward Solar-Driven Water Splitting. *ACS Nano* **14**, 5426–5434 (2020).
6. Chen, H. *et al.* Integrating Low-Cost Earth-Abundant Co-Catalysts with Encapsulated Perovskite Solar Cells for Efficient and Stable Overall Solar Water Splitting. *Adv. Funct. Mater.* **2008245**, 2008245 (2020).
7. Andrei, V. *et al.* Scalable Triple Cation Mixed Halide Perovskite–BiVO4 Tandems for Bias-Free Water Splitting. *Adv. Energy Mater.* **8**, 1801403 (2018).
8. Choi, H. *et al.* An organometal halide perovskite photocathode integrated with a MoS2catalyst for efficient and stable photoelectrochemical water splitting. *J. Mater. Chem. A* **9**, 22291–22300 (2021).
9. Kim, J. *et al.* Efficient and Stable Perovskite-Based Photocathode for Photoelectrochemical Hydrogen Production. *Adv. Funct. Mater.* 2008277 (2021). doi:10.1002/adfm.202008277
10. Ahmad, S. *et al.* Triple-Cation-Based Perovskite Photocathodes with AZO Protective Layer for Hydrogen Production Applications. *ACS Appl. Mater. Interfaces* **11**, 23198–23206 (2019).
11. Kim, I. S., Pellin, M. J. & Martinson, A. B. F. Acid-Compatible Halide Perovskite Photocathodes Utilizing Atomic Layer Deposited TiO 2 for Solar-Driven Hydrogen Evolution. *ACS Energy Lett.* **4**, 293–298 (2019).
12. Zhang, H. *et al.* A Sandwich-Like Organolead Halide Perovskite Photocathode for Efficient and Durable Photoelectrochemical Hydrogen Evolution in Water. *Adv. Energy Mater.* **8**, (2018).
13. Crespo-Quesada, M. *et al.* Metal-encapsulated organolead halide perovskite photocathode for solar-driven hydrogen evolution in water. *Nat. Commun.* **7**, 6–12 (2016).
14. Wang, M. *et al.* High-Performance and Stable Perovskite-Based Photoanode Encapsulated by Blanket-Cover Method. *ACS Appl. Energy Mater.* **4**, 7526–7534 (2021).
15. Kim, T. G. *et al.* Monolithic Lead Halide Perovskite Photoelectrochemical Cell with 9.16% Applied Bias Photon-to-Current Efficiency. *ACS Energy Lett.* **7**, 320–327 (2022).
16. Hoang, M. T., Phan, N. D., Han, J. H., Gardner, J. M. & Oh, I. Integrated

Photoelectrolysis of Water Implemented On Organic Metal Halide Perovskite Photoelectrode. *ACS Appl. Mater. Interfaces* **8**, 11904–11909 (2016).

17. Poli, I. *et al.* Graphite-protected CsPbBr3 perovskite photoanodes functionalised with water oxidation catalyst for oxygen evolution in water. *Nat. Commun.* **10**, 1–10 (2019).

18. Tao, R., Sun, Z., Li, F., Fang, W. & Xu, L. Achieving Organic Metal Halide Perovskite into a Conventional Photoelectrode: Outstanding Stability in Aqueous Solution and High-Efficient Photoelectrochemical Water Splitting. *ACS Appl. Energy Mater.* **2**, 1969–1976 (2019).

19. Nam, S., Mai, C. T. K., Oh, I., Thi Kim Mai, C. & Oh, I. Ultrastable Photoelectrodes for Solar Water Splitting Based on Organic Metal Halide Perovskite Fabricated by Lift-Off Process. *ACS Appl. Mater. Interfaces* **10**, 14659–14664 (2018).

20. Wang, C., Yang, S., Chen, X., Wen, T. & Yang, H. G. Surface-functionalized perovskite films for stable photoelectrochemical water splitting. *J. Mater. Chem. A* **5**, 910–913 (2017).

21. Reece, S. Y. *et al.* Wireless solar water splitting using silicon-based semiconductors and earth-abundant catalysts. *Science (80-.).* **334**, 645–648 (2011).

22. Fu, H. C., Varadhan, P., Lin, C. H. & He, J. H. Spontaneous solar water splitting with decoupling of light absorption and electrocatalysis using silicon back-buried junction. *Nat. Commun. 2020 111* **11**, 1–9 (2020).

23. Verlage, E. *et al.* A monolithically integrated, intrinsically safe, 10% efficient, solar-driven water-splitting system based on active, stable earth-abundant electrocatalysts in conjunction with tandem III–V light absorbers protected by amorphous TiO2 films. *Energy &EnvironmentalScience* **8**, 3166 (2015).

24. Sun, K. *et al.* A Stabilized, Intrinsically Safe, 10% Efficient, Solar-Driven Water-Splitting Cell Incorporating Earth-Abundant Electrocatalysts with Steady-State pH Gradients and Product Separation Enabled by a Bipolar Membrane. *Adv. Energy Mater.* **6**, 1–7 (2016).

25. May, M. M., Lewerenz, H. J., Lackner, D., Dimroth, F. & Hannappel, T. Efficient direct solar-to-hydrogen conversion by in situ interface transformation of a tandem structure. *Nat. Commun.* **6**, 4–10 (2015).

26. Okamoto, S., Deguchi, M. & Yotsuhashi, S. Modulated III-V triple-junction solar cell wireless device for efficient water splitting. *J. Phys. Chem. C* **121**, 1393–1398 (2017).

27. Young, J. L. *et al.* Direct solar-to-hydrogen conversion via inverted metamorphic multi-junction semiconductor architectures. *Nat. Energy* **2**, 1–8 (2017).

28. Kang, D. *et al.* Printed assemblies of GaAs photoelectrodes with decoupled optical and reactive interfaces for unassisted solar water splitting. *Nat. Energy 2017 25* **2**, 1–5 (2017).

29. Cheng, W. H. *et al.* Monolithic Photoelectrochemical Device for Direct Water Splitting with 19% Efficiency. *ACS Energy Lett.* **3**, 1795–1800 (2018).

30. Khaselev, O. & Turner, J. A. A monolithic photovoltaic-photoelectrochemical device for hydrogen production via water splitting. *Science (80-.).* **280**, 425–427 (1998).
