# Supplementary material for: Integrated halide perovskite photoelectrochemical cells with solar-driven water-splitting efficiency of 20.8%
Source: Nat Commun. 2023 Jun 26;14:3797. doi: 10.1038/s41467-023-39290-y (PMC10293190; doi:10.1038/s41467-023-39290-y)
Supplement: Supplementary file 2 — Description of Additional Supplementary Files [file 41467_2023_39290_MOESM2_ESM.docx]

Description of Additional Supplementary Files for

Integrated halide perovskite photoelectrochemical cells with solar-driven water-splitting efficiency of 20.8%

Austin M.K. Fehr, Ayush Agrawal, Faiz Mandani, Christian L. Conrad, Qi Jiang, So Yeon Park, Olivia Alley, Bor Li, Siraj Sidhik, Isaac Metcalf, Christopher Botello, James Young, Jacky Even, Jean Christophe Blancon, Todd G. Deutsch, Kai Zhu, Steve Albrecht, Francesca M. Toma, Michael Wong*, Aditya D. Mohite*

Correspondence to: adm4@rice.edu & mswong@rice.edu

**This file includes legends for:**

File Name: Supplementary Movie 1

Description: Hydrogen bubble formation on an illuminated photocathode surface, 16x speed.
